# 1000 Wh L^−1^ lithium-ion batteries enabled by crosslink-shrunk tough carbon encapsulated silicon microparticle anodes

**DOI:** 10.1093/nsr/nwab012

**Published:** 2021-01-23

**Authors:** Fanqi Chen, Junwei Han, Debin Kong, Yifei Yuan, Jing Xiao, Shichao Wu, Dai-Ming Tang, Yaqian Deng, Wei Lv, Jun Lu, Feiyu Kang, Quan-Hong Yang

**Affiliations:** Nanoyang Group, State Key Laboratory of Chemical Engineering, School of Chemical Engineering and Technology, Tianjin University, Tianjin 300072, China; Shenzhen Geim Graphene Center, Tsinghua Shenzhen International Graduate School, Tsinghua University, Shenzhen 518055, China; CAS Key Laboratory of Nanosystem and Hierarchical Fabrication, CAS Center for Excellence in Nanoscience, National Center for Nanoscience and Technology, Beijing 100190, China; Chemical Sciences and Engineering Division, Argonne National Laboratory, Argonne, IL 60439, USA; Nanoyang Group, State Key Laboratory of Chemical Engineering, School of Chemical Engineering and Technology, Tianjin University, Tianjin 300072, China; Nanoyang Group, State Key Laboratory of Chemical Engineering, School of Chemical Engineering and Technology, Tianjin University, Tianjin 300072, China; International Center for Materials Nanoarchitectonics (WPI-MANA), National Institute for Materials Science (NIMS), Tsukuba 305-0044, Japan; Shenzhen Geim Graphene Center, Tsinghua Shenzhen International Graduate School, Tsinghua University, Shenzhen 518055, China; Shenzhen Geim Graphene Center, Tsinghua Shenzhen International Graduate School, Tsinghua University, Shenzhen 518055, China; Chemical Sciences and Engineering Division, Argonne National Laboratory, Argonne, IL 60439, USA; Shenzhen Geim Graphene Center, Tsinghua Shenzhen International Graduate School, Tsinghua University, Shenzhen 518055, China; Nanoyang Group, State Key Laboratory of Chemical Engineering, School of Chemical Engineering and Technology, Tianjin University, Tianjin 300072, China; Joint School of National University of Singapore and Tianjin University, International Campus of Tianjin University, Fuzhou 350207, China

**Keywords:** lithium-ion batteries, anode, silicon microparticles, volumetric capacity, mechanical stability

## Abstract

Microparticulate silicon (Si), normally shelled with carbons, features higher tap density and less interfacial side reactions compared to its nanosized counterpart, showing great potential to be applied as high-energy lithium-ion battery anodes. However, localized high stress generated during fabrication and particularly, under operating, could induce cracking of carbon shells and release pulverized nanoparticles, significantly deteriorating its electrochemical performance. Here we design a strong yet ductile carbon cage from an easily processing capillary shrinkage of graphene hydrogel followed by precise tailoring of inner voids. Such a structure, analog to the stable structure of plant cells, presents ‘imperfection-tolerance’ to volume variation of irregular Si microparticles, maintaining the electrode integrity over 1000 cycles with Coulombic efficiency over 99.5%. This design enables the use of a dense and thick (3 mAh cm^–2^) microparticulate Si anode with an ultra-high volumetric energy density of 1048 Wh L^–1^ achieved at pouch full-cell level coupled with a LiNi_0.8_Co_0.1_Mn_0.1_O_2_ cathode.

## Teaser

An imperfection-tolerant carbon capsule cellular architecture well accommodates irregular microparticulate silicon anodes which represents a critical step forward in building compact lithium-ion batteries approaching ultrahigh volumetric energy density.

## INTRODUCTION

Silicon (Si) has been regarded as a high-energy replacement for graphite anodes due to its higher gravimetric (∼3579 mAh g^–1^) and volumetric-(>2000 mAh cm^–3^) specific capacities for Li_3.75_Si at room temperature [1–4]. Especially, microparticulate Si (SiMP) forms an attractive electrode material with lower cost and a much higher tap density than Si nanoparticles [5–7] (SiNPs, Fig. S1). More promisingly, with a lower surface area, interfacial issues such as electrolyte decomposition and thermal risks can be largely avoided (Fig. S1e) [8]. However, SiMPs larger than a critical size of 150 nm are subject to structural pulverization during cycling, resulting in electrical disconnection and continuous consumption of solid electrolyte interphases (SEIs) [2]. Self-healing binders or electrolyte designs have been used to maintain the particle integrity of SiMPs to improve their cycling performance, which is still challenged by its limited effect on stabilizing the SEI layer to survive repeated volume changes during a long cycle life (>500 cycles) [9–12].

**Figure 1.**
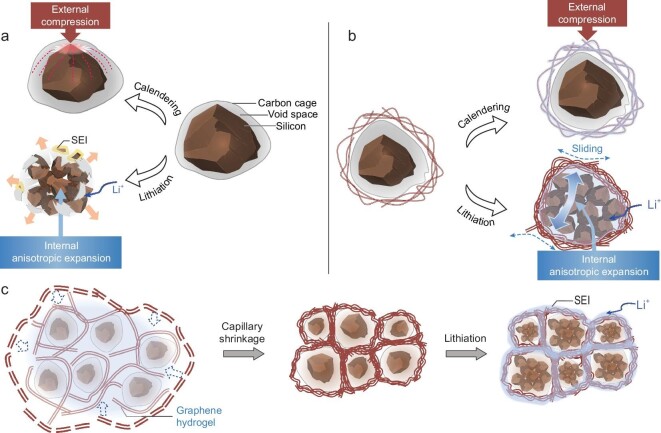
Schematic of a highly imperfection-tolerant 3C-architecture design for microparticulate Si anodes. (a) SiMP@C, namely SiMPs in a highly graphitic carbon cage (normally prepared with a CVD procedure) with the predefined void that buffers pulverized Si nanoparticles. Such a SiMP@C is always ruptured under the high-pressure compression during calendering and the anisotropic expansion of irregular-sized SiMPs in electrochemical cycling. The exposed fresh Si surface induces severe side reactions, accumulating new SEI. (b) SiMP@C-GN, where the carbon cage (‘cell membrane’) is further stabilized and protected by a highly dense and robust graphene shell (‘cell wall’) inspired by the superior structural stability of a plant cell. The densely entangled graphene sheets can withstand the external compression, whereas their interlayer-sliding endows the imperfection-tolerance ability to manage the internal anisotropic expansion. (c) The formation of 3C architecture in SiMP@C-GN. SiMP@C is interwoven into the 3D graphene network with a hydrothermal process and the following capillary shrinkage (∼20 × volume shrinkage) allows crumple and densified graphene network tightly adhered to the surface of SiMP@C to yield the final SiMP@C-GN with dense and robust 3C architecture, forming a mechanically and electrically integrated structure with good imperfection-tolerance to repetitive volume variation during cycling.

High-quality carbon coating with preserved voids on SiMPs to contain Si debris and protect their surface against electrolyte interaction is an effective strategy [7,13–15]. Nevertheless, in real working conditions as well as the cycling process, carbon coating can be easily fractured as illustrated in Fig. 1a: (i) it is practically difficult to preserve an appropriate void volume compatible with SiMPs of irregular sizes and anisotropic properties [16–18]; (ii) with severe volume changes of SiMPs, cracks will likely be generated, leading to the failure of the whole electrode; (iii) the micro-size void between carbon shells and SiMPs could hardly withstand the high calendaring pressure (>80 MPa) during electrode fabrication [19]. Previously reported carbon encapsulation could not simultaneously satisfy the stringent mechanical requirements during fabrication and cycling. For example, graphitic carbon coating is strong but less ductile, and tends to be fractured by the internal tensile stress [20]. On the other hand, the loosely porous graphene network is easily deformed and could not keep stability under compression [21].

Here we design an imperfection-tolerant unique carbon capsule cellular (3C) architecture, which consists of carbon cages with rational voids interweaved in a cellular dense graphene network, to effectively overcome the conflicts of strength and ductility. Inspired by the superior structural stability of plant cells over animal cells (free of cell walls) (Fig. 1b), a highly dense and interlinked graphene network (works as ‘cell wall’) tightly adheres to the surface of the chemical vapor deposition (CVD) carbon (‘cell membrane’)-caged SiMPs with predefined voids (‘cytoplasm’), forming a mechanical and electrical integrity due to a capillary shrinkage process of graphene hydrogel (Fig. 1c). The 3C architecture has impressive strength and ductility, which could overcome the mechanical challenges at multiple length scales from individual particle to electrode. In particular, the sliding characteristics of highly curved and interlinked graphene sheets endow the imperfection-tolerance ability to manage stress and spatial distribution during cycling. Consequently, a record long life of 1000 cycles with average Coulombic efficiency (CE) over 99.5% is achieved in lithium-ion batteries using this SiMP anode. Because of the dense carbon architecture and the retained high density of SiMPs, the anode exhibits a very high volumetric capacity (∼1500 mAh cm^–3^). At the pouch full-cell level, coupling such SiMP anode with a LiNi_0.8_Co_0.1_Mn_0.1_O_2_ (NCM811) cathode, an ultra-high volumetric energy density of 1048 Wh L^–1^ has been achieved. Moreover, under a high areal capacity of 2 mAh cm^–2^, the SiMP@C-GN/NCM811 full-cell is stable up to a long 200-cycle life, which is the best published result to date for the solid SiMP anodes in full-cell tests.

## RESULTS AND DISCUSSION

### Synthesis of the 3C encapsulated SiMPs

**Figure 2.**
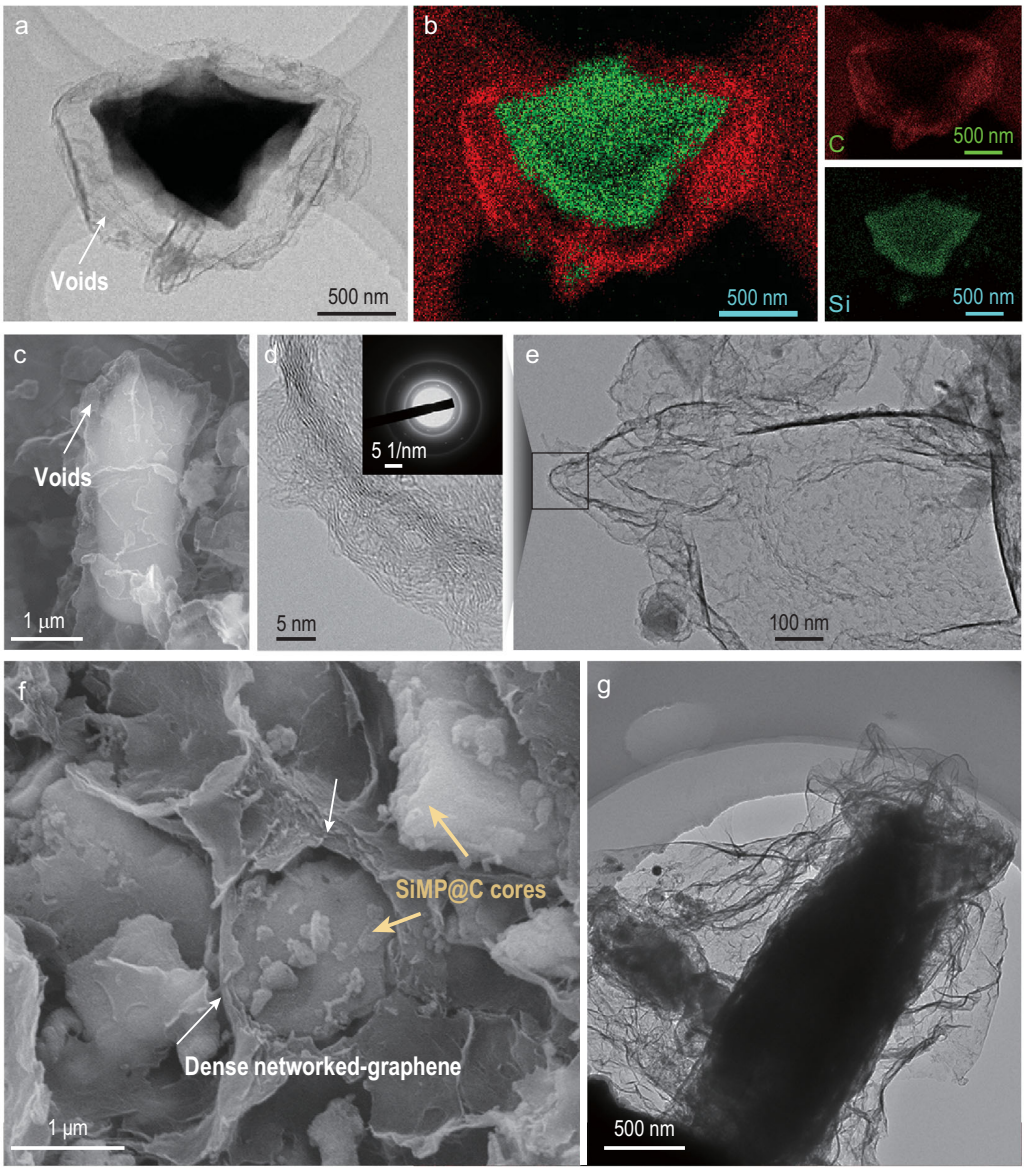
Morphology and structural characterization of the carbon encapsulation. (a) TEM image of a SiMP@C. (b) TEM-EDS elemental maps of the SiMP@C in (a), showing the hollow structure and the conformal carbon cage. (c) SEM image of a SiMP@C. (d and e) TEM images of SiMP@C after the total etching removal of Si. Inset image in (d) shows an SAED pattern of the carbon cage. (f) SEM image of SiMP@C-GN, which reveals the plant cell tissue-like structure. (g) TEM image of SiMP@C-GN.

Firstly, a graphitic carbon shell was coated on each SiMP by CVD with a CH_4_ source. An appropriate amount of void structures inside the carbon shell were then produced by a well-controlled NaOH etching process, and they could allow expansion of the Si upon lithiation (SiMP@C, Fig. S2a–c). One key feature of this robust SiMP anode is the building of a high-quality carbon cage around the particles. Transmission electron microscopy (TEM) (Fig. 2a) and energy-dispersive X-ray spectroscopy (EDS) maps (Fig. 2b) demonstrate the voids in the SiMP@C hybrid microparticles. The volume of void space and the mass of Si in this hybrid can be controlled by changing the NaOH etching time (Fig. S4). As shown in the scanning electron microscopy (SEM) image (Fig. 2c), a thin carbon cage with voids is constructed surrounding the SiMPs. A broader view of SiMP@C microparticles (Fig. S5a) shows the etched void space is relatively uniform. The etched depth follows a normal distribution, mainly in the range of 600–800 nm, showing the overall homogeneity of the NaOH etching technique (Fig. S5b). With total etching of the Si component, the carbon cage remains robust, and is highly graphitic (Fig. 2d and e). The selected area electron diffraction (SAED) pattern in the inset of Fig. 2d and the G and 2D peaks in the Raman spectrum confirm a graphitic characteristic of the CVD-grown carbon (Fig. S6). In addition, there are some defects in the graphitic structure indicated by the D band in the Raman spectrum (Fig. S6a), which permit NaOH to etch inner Si during material synthesis and facilitate Li^+^ transport during battery operation.

Secondly, to obtain the 3C architectures, SiMP@C was interwoven into the interlinked graphene hydrogel during a hydrothermal process (Fig. S2d). Subsequently, the contraction of hydrogel is induced by capillary evaporation, driven by the surface tension of trapped water, which has a high surface tension and thus exerts a strong capillary force on the graphene nanosheets to achieve a huge shrinkage (Movie S1) [22,23]. As a result, the crumple and densified graphene network (like ‘cell wall’) tightly adheres to the surface of SiMP@C to yield the final product (SiMP@C-GN, Fig. S2e). As shown in Fig. 2f and g and Fig. S7, the hydrogel shrinks into a dense monolith (∼20 × volume shrinkage), inside which the graphene network tightly contracts on the surface of the dispersed particles, forming a dense buffer layer, and incorporating the particles into a strong and electrically conductive integrity, that is, the 3C architectures. Notably, due to the low-cost raw materials as well as the high scalability of the entire fabrication process (Fig. S3), we can implement a simple, clean and continuous production, demonstrating the potential of this method for scalable production.

### Study on mechanical properties of SiMP@C-GN

**Figure 3.**
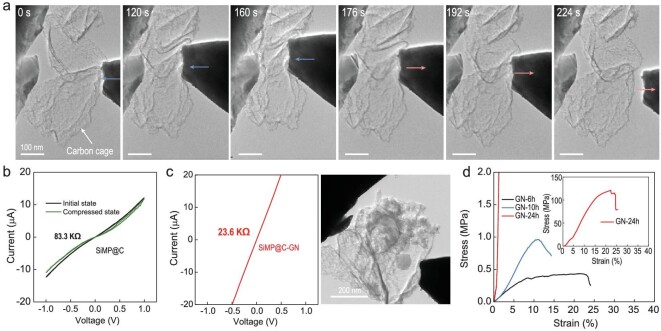
Electrical and mechanical property characterization of SiMP@C and SiMP@C-GN. (a) *In situ* TEM observations of the inner carbon cages under compressive deformation. (b) Current-voltage curves of the carbon cage. (c) Current-voltage curve of the SiMP@C-GN structure shown in the TEM image on the right. (d) Stress-strain curves of the compression of GN-24h, -10h and -6h cylinders. Inset is the stress-strain curve for GN-24h in full range.

*In situ* TEM experiments were conducted to test the electrical and mechanical properties of the inner carbon cage. Under the indentation and compression by a piezo-controlled probe, the carbon cage structure showed good mechanical strength and flexibility so that it could endure severe deformation. When the compressing probe was withdrawn, the original structure was gradually restored (Fig. 3a, Movie S2). The high mechanical strength and flexibility of the inner carbon cage endows the 3C-architecture encapsulation with the first-step buffer to accommodate the SiMP expansion in the early lithiation process. The cage has a high electrical conductivity because of its highly graphitic structure (83.3 kΩ, Fig. 3b) and this shows little change under deformation.

In the 3C architecture, the highly curved and interconnected graphene sheets stick tightly to the surface of the inner carbon cage producing good electrical connectivity with the individual SiMP@C cores, which thus guarantees fast electron supply to the inner SiMPs during charge/discharge. This structure presents a resistance of 23.6 kΩ (Fig. 3c), which is much lower than that of the inner carbon cages. To demonstrate the mechanical reinforcement provided by such a dense network of graphene, its mechanical properties (samples denoted GN-X, where X is the capillary drying time in hours) were evaluated by measuring compression properties of the fabricated graphene macroform. The stress-strain curves show that with larger capillary shrinkage and full densification, the mechanical properties are dramatically enhanced [21], with the compressive strength up to ∼121 MPa, and the fracture toughness (defined as the area under the stress-strain curve) up to ∼1.3 × 10^7^ J m^–3^, respectively (Figs 3d and S8; Table S1). It is noted that for the GN-6h sample, yielding at ∼8% is observed in the stress-strain curve, corresponding to the cracking of the graphene framework. However, it is not catastrophically fractured due to the lower density and looser structure. After yielding, the GN-6h sample is densified by the compression and therefore the stress-strain curve is further extended with a higher pressure loading until a 25% strain. The significant improvement of the mechanical properties of dense GN-24h compared to that of loosely structured GN-10h and -6h can be ascribed to the density scaled mechanical strength. Also, the highly interlinked graphene sheets allow for interlayer-sliding, affording a high ductility at the same time. In our recent research, with a sound size-scaling effect, this compact graphene network in a form of micropillar showed a high yield strength of over 200 MPa and a yield strain of 58%, and even showed a high strength (1.57 GPa) and ductility (15%) in nanopillars [24]. Even when cracks were initiated in the monolith, the stress concentration was dissipated through the graphene network to avoid fast crack propagation and catastrophic failure. The above results show that this continuous and dense network of graphene is remarkably robust and is able to withstand external loads with a high strength and internal stresses with a high ductility, which endows 3C architecture a high imperfection-tolerance ability to survive the calendering and buffer the anisotropic expansion of the irregular-sized SiMPs.

### Electrochemical performance of SiMP@C-GN

**Figure 4.**
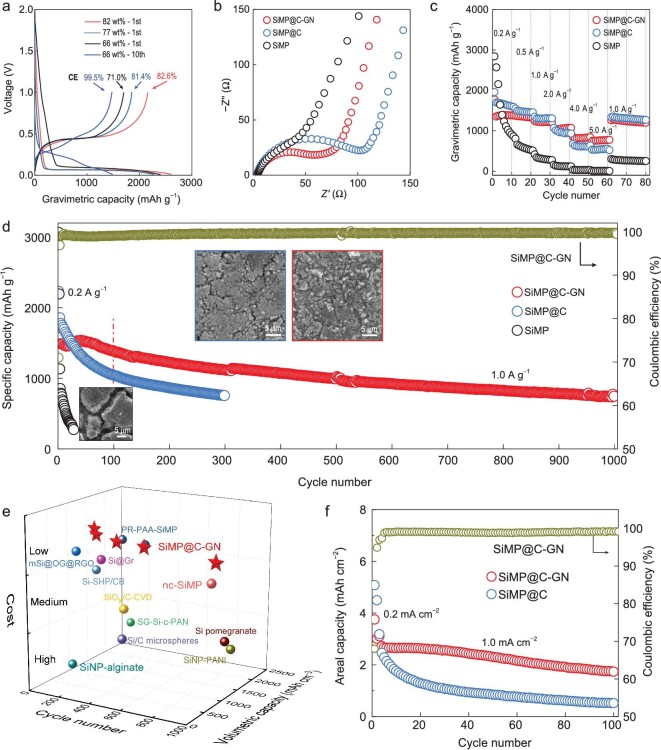
Electrochemical characterization of 3C-architecture encapsulated SiMP anodes. (a) Optimized initial Coulombic efficiency of SiMP@C-GN with different Si contents. (b) EIS measurements of SiMP@C-GN (Si mass content is 66 wt%), SiMP@C and bare SiMP electrodes in their original states. (c) Rate capabilities of SiMP@C-GN (Si mass content is 66 wt%), SiMP@C and bare SiMP electrodes at current densities of 0.2, 0.5, 1.0, 2.0, 4.0 and 5.0 A g^–1^. (d) Half-cell discharge capacities of a SiMP@C-GN anode compared with the control samples (SiMP@C and bare SiMP anodes) tested under the same conditions. The charge/discharge current density is 0.2 A g^–1^ for the first three cycles and 1.0 A g^–1^ for later cycles. The inset SEM images show the morphology changes of SiMP, SiMP@C and SiMP@C-GN (Si mass content is 66 wt%) electrodes after 100 cycles. (e) Comparison of the SiMP@C-GN anode with other reported anodes (PR-PAA-SiMP [25], Si@Gr [7], mSi@OG@RGO [13], nc-SiMP [26], SiO*_x_*/C-CVD [27], Si-SHP/CB [28], SiNP-PANi [29], Si pomegranate [30], SG-Si-c-PAN [31], SiNP-alginate [32], Si/C microspheres [33]) in terms of estimated processing cost, number of cycles and volumetric capacity. (f) Cycling performance of high-mass-loaded SiMP@C-GN and SiMP@C anodes (equal initial areal capacity at 1.0 mA cm^–2^). All electrodes were first cycled at 0.2 mA cm^–2^ for the first three cycles and 1.0 mA cm^–2^ for later cycles.

To verify the rationality of the design strategy proposed in Fig. 1, the electrochemical performance of SiMP, SiMP@C and SiMP@C-GN was evaluated in half-cell and full-cell configurations. The coin-type half cells were assembled with lithium (Li) as the counter electrode, with the voltage ranging from 0.01 to 1.00 V. All calculated capacities are based on the total mass of electrodes, including Si, C and all other inactive components except the current collector. The galvanostatic charge/discharge voltage profiles in Fig. 4a show a high initial Coulombic efficiency (ICE) of the SiMP@C-GN electrode (up to 82.6%) for 82 wt% Si content. The dense graphene network helps the inner carbon-caged SiMPs to deliver superior cyclability (Fig. S9). With a trade-off of the capacity (Si content), cycling performance (buffering effect) and rate performance (electrical conductance), the SiMP@C-GN anode with 66% Si content was chosen to demonstrate the 2-fold buffering strategy for the SiMPs (Fig. S10). The CE of a SiMP@C-GN anode containing 66% Si rapidly increased to 99.5% after 10 cycles. To address the concerns of SiMP anodes in cycling reversibility, the defects on carbons and their electrical conductivity are further optimized by thermal annealing (1000°C), as a result of which the ICE is enhanced up to 84.5% (Fig. S11). Also, pre-lithiation techniques are employed to compensate for the initial lithium loss to a larger extent, which is demonstrated in the full-cells. According to Nyquist plot obtained from electrochemical impedance spectroscopy (EIS), the SiMP@C-GN electrode has the smallest charge-transfer resistance, as evidenced by the diameter of the semicircle in the high-frequency region (Fig. 4b). This result indicates that among the three SiMP electrodes, the charge-transfer resistance of SiMP@C-GN anode is the smallest, which is attributed to the continuous and dense graphene network that improves electrical contact and facilitates electron transfer from the graphene to the surface of each carbon-caged microparticle. In addition, the SiMP@C-GN anode shows superior rate performance at large current densities ranging from 2.0 to 5.0 A g^−1^ compared to the SiMP@C anode (Fig. 4c), confirming the faster charge transport in the 3C architectures.

Figure 4d shows the cycling performance of the three SiMP anodes, where the bare SiMP anode shows a rapid capacity decay with less than 400 mAh g^–1^ after only 20 cycles. With the good mechanical flexibility and electrical conductivity of the graphitic carbon cage, the cyclic stability of SiMP@C is significantly improved compared to that of the bare SiMP anode. Nevertheless, the capacity decay of SiMP@C is apparent in the early cycles as its capacity retention is only 63% after the initial 100 cycles, which could be ascribed to the unavoidable volume expansion and even rupture of the SiMP@C hybrid microparticles as the SiMPs become pulverized upon repeated lithiation. In contrast, the SiMP@C-GN anode shows superior cycling stability to the SiMP@C anode, especially in the first 100 cycles (92.5%). A high 70% capacity retention (1050 mAh g^–1^) was achieved after 500 cycles. Even 774 mAh g^–1^ of capacity remained after 1000 cycles at a current density of 1.0 A g^–1^, which greatly exceeds that of SiMP@C and is twice that of the theoretical capacity of commercial graphite anodes. To the best of our knowledge, this is the longest cycling life achieved in a SiMP-based anode (Table S2). Also, because of the high density of SiMP@C-GN, the volumetric capacity based on the whole electrode reaches 1386 mAh cm^–3^ after 100 cycles. With the processing cost, volumetric capacity and cycling life taken into account, the performance of the SiMP@C-GN anode is among the best published to date for Si-based anodes, including SiMP, sub-SiMP and SiNP anodes (Fig. 4e) [7,13,25–33]. The excellent cycling performance of SiMP@C-GN can be mainly attributed to the buffering of the CVD carbon and the dense graphene network, which together improve the structural stability of the electrode and produce a high-efficiency utilization of the active SiMPs. After cycling, the pulverized Si particles are still well encapsulated and protected by intact 3C architectures from electrolyte contacts (Figs S12a and S13). Furthermore, high-magnification TEM and EIS analyses (Fig. S12b and c) of the SiMP@C-GN after cycling show that the pulverized Si particles are electrically connected, which ensures a high utilization of SiMPs in the SiMP@C-GN anode during the long cycle life to deliver a high gravimetric capacity. Further interfacial reaction between pulverized SiMPs and electrolytes and the accumulation of SEI are also greatly alleviated. This can be evidenced by Fig. S14. Specifically, for the pulverized Si particles, the carbon still has an intact covering on them. Moreover, the F components contained in the SEI layer have a distribution similar to the carbon elements, without an enrichment on the areas of Si particles. The surface morphology change of the three SiMP anodes was also investigated after repeated cycles by top-view SEM (inset of Fig. 4d and Fig. S15). Before cycling, all three anodes have a flat and smooth surface (Fig. S15a–c). However, after 100 cycles, severe cracks and obvious particle disintegration occur in the bare SiMP electrode (Fig. S15d and g), leading to the loss of electrical connection in the electrode with subsequent SEI formation on the newly exposed Si surfaces, which account for the rapid capacity decay. Even though these problems are alleviated in a SiMP@C electrode with the carbon cage protection, many cracks can still be observed in Fig. S15e. High-magnification SEM images in the inset of Fig. 4d and Fig. S15h show that several active particles are separated from the electrode due to the repetitive volume expansion and contraction during cycling, forming distinct cracks between the electrode components. This verifies that a single carbon cage is inadequate to withstand the volume change of SiMPs during cycling. As for the SiMP@C-GN anode, there are no obvious cracks after 100 cycles (inset of Fig. 4d; Fig. S15f and i). The active hybrid electrode maintains mechanical integrity as well as electrical connection, which is a result of the highly curved and interlocked graphene cellular structure that effectively absorbs the local stresses to avoid extensive crack propagation in the electrode.

To elucidate the synergistic effect of the SiMP@C-GN composite structure, different encapsulation designs were used in SiMP anodes. Specifically, to demonstrate the significance of the inner carbon cage and void space, a comparison of cycling stability was made between the three types of Si-based materials with similar carbon contents (Fig. S16). One is SiMPs directly encapsulated by a dense graphene network after capillary drying (denoted as SiMP-GN) and the other two are SiMP@C-GN with/without etching (Fig. S17). It is obvious that SiMP@C-GN without void etching shows superior cycling performance (75.2% capacity retention for the first 100 cycles) compared to SiMP-GN (67.0%), indicating that the inner carbon cage protects the cracked SiMPs against electrolyte decomposition and thus stabilizes the SEI layer on their surface. Voids inside the carbon cage produce a further improvement in performance (92.5%) compared to the SiMP@C-GN without etching, demonstrating that void space in the carbon cage is indispensable in accommodating the volume change of the SiMPs.

To investigate the feasibility of SiMP@C-GN for practical application, electrodes with a high mass loading of Si were further tested in half-cells. It can be seen in Fig. 4f that the capacity difference between SiMP@C-GN and SiMP@C anodes increases with electrode thickness during cycling. For SiMP@C-GN with a mass loading of 2.4 mg cm^–2^, the initial reversible areal capacity reached 2.7 mAh cm^–2^ at a current density of 1 mA cm^–2^ and remained at 1.8 mAh cm^–2^ after 100 cycles. In contrast, the SiMP@C electrode with the same initial reversible areal capacity at 1 mA cm^–2^ showed poorer cycling performance with only ∼0.5 mAh cm^–2^ remaining after 100 cycles. A supplementary cycling test with an equal initial discharging capacity at an active current density of 0.2 mA cm^–2^ for SiMP@C and SiMP@C-GN is further performed (Fig. S18), which demonstrates a similar trend with a distinct gap of cycling stability between them. This result indicates that the 3C architectures certainly provided excellent structural and electrical stability even with a large electrode thickness. Figure S19 shows a cross-sectional SEM image of the thick electrodes composed of SiMP@C and SiMP@C-GN before and after cycling. The SiMP@C anode with a high-mass loading undergoes an obvious thickness increase of 167% after 50 cycles, while the thickness increase is limited to 57% for SiMP@C-GN, which is acceptable for Si-based electrodes in practical battery applications. Moreover, the SiMP@C particle after 50 cycles shows a relatively rough SEI layer on the surface due to the repeatedly exposed active Si surface upon lithiation (Fig. S20a and b). As a comparison, the SiMP@C-GN particle has an intact and dense encapsulation for SiMPs, and the SEI formed on the surface is smooth and thin (Fig. S20c and d).

### *In situ* lithiation of SiMP@C-GN

**Figure 5.**
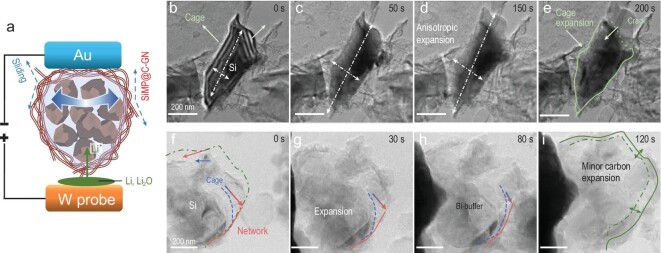
*In situ* TEM observation of SiMP volume expansion upon lithiation. (a) Schematic of the micro-cell for *in situ* lithiation. (b−e) Lithiation of SiMP@C. Because the volume expansion is anisotropic, the carbon cage expands and is finally ruptured by the expansion of the Si. Eventually the SiMP@C has a large expansion. (f−i) Lithiation of SiMP@C-GN. Despite severe anisotropic expansion of the SiMPs, the surrounding carbon and graphene effectively buffer the volume change and maintain the SiMP protection.

To further evaluate the buffering effect, *in situ* TEM electrochemical tests were performed to observe the lithiation behavior of SiMP@C and SiMP@C-GN. An electrochemical micro-cell was assembled with a gold (Au) rod as the working electrode and a small piece of Li coated with lithium oxide (Li_2_O) attached to the tip of a tungsten (W) probe as the counter electrode (Fig. 5a). SiMP@C or SiMP@C-GN active material was loaded on the edge of the Au electrode and TEM images of the active material were recorded during lithiation (Movies S3 and 4). When the Li/Li_2_O is pushed into electrical contact with the active material, the Si particles begin to expand. With continuous Li-ion insertion, the SiMP in the carbon cage expands slowly (Fig. 5b–e and Movie S3). Because of the anisotropic crystal structure and mechanical properties, the volume deformation of the Si in SiMP@C is non-uniform (Fig. 5d). When the SiMP expands and fills the whole void space, the carbon cage also expands and eventually cracks (upper region of Fig. 5e). In contrast, despite the dramatic expansion of the internal SiMP, the SiMP@C-GN material is robust enough to withstand the anisotropic volume expansion of the SiMP (Fig. 5f–h and Movie S4). In the final lithiated state of SiMP@C-GN, its structural integrity survives with a minor volume expansion (∼36%) with the stress effectively relaxed by the interlayer-sliding of the graphene cellular network (Fig. 5i). Therefore, compared to the sample of SiMP@C, SiMP@C-GN exhibits the ability of stress management of the efficient stress dissipation to successfully address the anisotropic expansion of Si microparticles, i.e. ‘imperfection-tolerance’. Besides, considering the possible constraint effect on the lithiated Si conversely from the mechanically-tough 3C architectures [34], it is critical to incorporate an appropriate amount of void space in the CVD carbon cage, together with optimized content of dense graphene network in this composite to provide suitable interlayer-sliding adaptable to the expansion rate of Si and retain the mechanical stability of anodes at the same time.

### Practicability of SiMP@C-GN anodes

**Figure 6.**
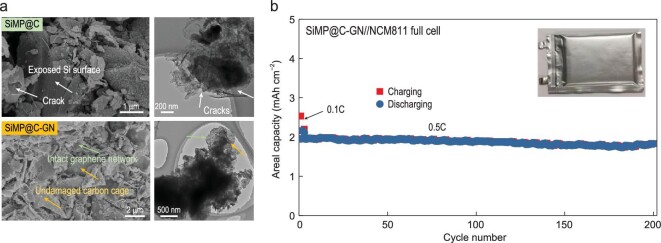
Practicability of SiMP@C-GN in anode fabrications and full-cell tests. (a) SEM and TEM images of SiMP@C and SiMP@C-GN electrodes after practical pressure pressing (80 MPa). (b) Cycling performance of the SiMP@C-GN/NCM811 pouch full-cell with a high areal capacity over 2 mAh cm^−2^. The charge/discharge current density is 0.1C for the first two cycles and 0.5C for later cycles (1C = 190 mA g^−1^ for the NCM811 cathode).

Furthermore, to evaluate the potential of the SiMP@C-GN anode materials in practical devices, a coin full-cell test was performed. First, fracture during calendering in the practical electrode fabrication process must be avoided. Thus, the structural stability under realistic pressure was also evaluated for the SiMP@C and SiMP@C-GN anodes. With a high rolling pressure of 80 MPa, the SiMP@C electrode suffered cracking of the carbon cages, resulting in exposure of the Si surface (Fig. 6a). In comparison, SEM and TEM observations of the compressed SiMP@C-GN anode showed a stable protection layer, comprised of the intact graphene network and undamaged carbon cages for the inner SiMPs, showing the excellent pressure resistance of this 3C architecture with the dense network of graphene layers. Second, a coin full-cell test using lithium cobalt oxide (LCO) as the cathode was first performed. With an electrochemical pre-lithiation process, the full-cell with a 2.5–4.2 V operating voltage window delivered a high initial Coulombic efficiency of 94%, higher than that of commercial graphite anodes in lithium-ion batteries. The SiMP@C-GN/LCO full-cell also delivered excellent Coulombic efficiency, reaching 99.4% after the first seven cycles, and demonstrated a stable cycling performance (102 mAh g^–1^ after 100 cycles based on the mass of LCO) (Fig. S21). To demonstrate the possibility of achieving a high volumetric energy density based on the practical areal capacity (3 mAh cm^–2^), we also assembled a SiMP@C-GN/NCM811 pouch full-cell (5.3 cm × 6.7 cm, 35.51 cm^2^) with an average voltage of 3.6 V and a total thickness of 103 μm (including electrodes, separator, electrolyte filled into electrodes and current collectors) (Fig. S22). This full-cell delivered 1048 Wh L^–1^ after 50 cycles, which is much higher than that of commercial lithium-ion batteries (550 Wh L^–1^) [35]. To the best of our knowledge, this is among the highest volumetric energy density reported for lithium-ion batteries (Table S3) [36]. Under an areal capacity of 2 mAh cm^–2^, the SiMP@C-GN/NCM811 full-cell is stable up to 200-cycle life (Fig. 6b), which is the best published result to date for the solid SiMP anodes in full-cell tests (Table S4). Even at a high current density of 1C, the full-cell with SiMP@C-GN anodes still delivers good cyclic stability (Fig. S23). We also tested a 1.2 Ah pouch full-cell with a six-layer assembly and realistic electrolyte usage based on a combination of the SiMP@C-GN anode and a NCM811 cathode. The reversible specific capacity reaches 171 mAh g^–1^ with good cyclic stability, demonstrating the applicable future of SiMP@C-GN anode materials in lithium-ion batteries (Fig. S24).

## CONCLUSION

To have a practically applicable SiMP anode with low specific surface area and high tap density, we report here an imperfection-tolerance design using 3C architectures to accommodate the volume change of SiMPs during cycling. In this design, the capillary shrinkage driven by the surface tension of the trapped solvent is employed to interweave a continuous and dense graphene network on the surface of carbon-caged SiMPs, giving the electrode excellent mechanical and electrical robustness. Such a 3C architecture with strong carbon cages in the graphene network is demonstrated as a highly desirable hybrid structure for SiMP anodes. As a result, lithium-ion batteries with an ultra-long life of 1000 cycles together with both high gravimetric and volumetric capacity are achieved. Also, with a high mass loading, a pouch full-cell with a SiMP@C-GN anode and a NCM811 cathode gives an ultra-high volumetric energy density of 1048 Wh L^–1^. Under an areal capacity of 2 mAh cm^–2^, the SiMP@C-GN/NCM811 full-cell is stable up to 200 cycles. With an intact surface protection to reduce the side reactions and a mechanically tough framework to dissipate local high stress, the volume expansion and particle pulverization issues in SiMP anodes during battery cycling have been significantly mitigated. Effective use of the low-cost, low-surface-area and high-tap-density SiMP anode is promising to enable its practical applications in lithium-ion batteries considering the successfully suppressed interfacial issues related to electrolyte consumption and safety concern. Moreover, the novel mechanical properties with both high strength and toughness in this capillary shrunk graphene network can be quite useful to mitigate the structural instability issues of beyond-Si electrodes suffering similar volumetric variation upon Li^+^ storage/release, such as Ge, Sn, Al anodes and S, Te cathodes.

## METHODS

### Synthesis of SiMP@C and SiMP@C-GN

SiMP@C was prepared by a CVD method together with partial etching of the Si by sodium hydroxide (NaOH). Specifically, the used crystalline SiMP powders have particle size in the range of 3–5 μm (Fig. S1b–d). Then, the CVD carbon deposition on SiMP powder was purged with argon (Ar) gas (50 mL min^−1^) and subsequently CH_4_ (50 mL min^−1^) gas when heated to 1000°C for 60 min. The above carbon-coated SiMPs were then etched by NaOH at 70°C to obtain SiMP@C. To prepare the SiMP@C-GN, the SiMP@C powders dispersed in ethanol were mixed with the GO powder, prepared through the well-known modified Hummers method, to form a suspension. Afterwards, this suspension was sealed in a 100 mL Teflon-lined autoclave and heated at 180°C for 6 h to obtain a cylindrical hybrid hydrogel. Finally, the capillary drying technology was employed to shrink this hydrogel into a dense monolith (∼20 × volume contraction), yielding the SiMP@C-GN product. Specifically, the hydrogel was subjected to an evaporation-induced drying at 70°C for 48 h under air and atmospheric pressure [22,23,37].

### Material characterizations

Thermogravimetric analysis (TG, Rigaku, Japan) was performed at a heating rate of 10°C min^–1^ from room temperature to 1000°C under an air atmosphere to calculate the Si contents. SEM observations were performed on a Hitachi S-4800 (Hitachi, Japan). Phase purity and crystal structure were characterized by X-ray diffraction (XRD) (Bruker D-8 diffractometer, Cu Kα radiation, *λ* = 0.154 nm). TEM images were collected on a JEM 2100F (JEOL, Japan) operated at 200 kV and equipped for EDS for elemental analysis. *In situ* TEM experiments were performed on a JEOL-3100 FEF, which is equipped with a Nanofactory Instruments STM-TEM holder.

### Electrochemical measurements

The anode was composed of SiMP-based active materials and binders of polyaniline (PANi). The electrode density was ∼1.0 g cm^–3^. The total mass loadings (including active materials and binders) were ∼1.0 mg cm^–2^ (∼1.5 mAh cm^–2^) for relatively thin electrodes and ∼2.3 mg cm^–2^ (∼3 mAh cm^–2^) for thick electrodes, respectively.

For the half-cell test, lithium metal foil was used as the counter electrode and 1 M lithium hexafluorophosphate (LiPF_6_) in 89 vol% ethylene carbonate/diethyl carbonate (EC/DEC) (1 : 1, v/v) with 10 vol% fluoroethylene carbonate (FEC) and 1 vol% vinylene carbonate (VC) was used as the electrolyte. For the full-cell test, the SiMP@C-GN was paired with an LCO or an NCM811 cathode with an N/P ratio of ∼1.1. The battery performance was evaluated by galvanostatic charge/discharge measurements conducted on a LAND testing system at room temperature with 0.01–1 V for the half-cell and 2.5–4.2 V for the full-cell versus Li^+^/Li. EIS was performed with an alternating current voltage amplitude of 5 mV in the frequency range of 100 kHz to 0.01 Hz using an electrochemical workstation (Metrohm Autolab, Switzerland).

## Supplementary Material

nwab012_Supplemental_Files
