# Supplementary material for: 1000 Wh L−1 lithium-ion batteries enabled by crosslink-shrunk tough carbon encapsulated silicon microparticle anodes
Source: Natl Sci Rev. 2021 Jan 23;8(9):nwab012. doi: 10.1093/nsr/nwab012 (PMC8433081; doi:10.1093/nsr/nwab012)
Supplement: nwab012_Supplemental_Files [file nwab012_supplemental_files.zip › Supplementary_data.docx]

**1000 Wh L^-1^ lithium-ion batteries enabled by crosslink-shrunk tough carbon encapsulated silicon microparticle anodes**

Fanqi Chen^1,†^, Junwei Han^2,†^, Debin Kong^3,†^, Yifei Yuan^4^, Jing Xiao^1^, Shichao Wu^1^, Dai-Ming Tang^5,^ ,^*^ Yaqian Deng^2^, Wei Lv^2^, Jun Lu^4,*^ , Feiyu Kang^2^ and Quan-Hong Yang^1,6,*^

^1^Nanoyang Group, State Key Laboratory of Chemical Engineering, School of Chemical Engineering and Technology, Tianjin University, Tianjin 300072, China

^2^Shenzhen Geim Graphene Center, Tsinghua Shenzhen International Graduate School, Tsinghua University, Shenzhen 518055, China

^3^CAS Key Laboratory of Nanosystem and Hierarchical Fabrication, CAS Center for Excellence in Nanoscience, National Center for Nanoscience and Technology, Beijing 100190, China

^4^Chemical Sciences and Engineering Division, Argonne National Laboratory, Argonne, IL 60439, USA

^5^International Center for Materials Nanoarchitectonics (WPI-MANA), National Institute for Materials Science (NIMS), Tsukuba 305-0044, Japan

^6^Joint School of National University of Singapore and Tianjin University, International Campus of Tianjin University, Fuzhou 350207, China

†Equally contributed to this work.

***Corresponding authors.** E-mails:

[tang.daiming@nims.go.jp](mailto:tang.daiming@nims.go.jp); [junlu@anl.gov](mailto:junlu@anl.gov); [qhyangcn@tju.edu.cn](mailto:qhyangcn@tju.edu.cn)

**Supplementary experimental methods**

**1. Synthesis of SiMP@C**

SiMP@C was prepared by a CVD method together with partial etching of the Si by sodium hydroxide (NaOH). 1.0 g of crystalline SiMP powder with the particle size in the range of 3–5 μm (Fig. S1b−d) was placed in a horizontal quartz tube. The tube was first purged with high-purity argon (Ar) gas (50 mL min^−1^) and then heated to 1000°C at a heating rate of 10°C min^−1^. CH_4_ gas was then introduced with a flow rate of 50 mL min^−1^. After CVD at 1000°C for 60 min, the CH_4_ gas was cut off and the system was cooled to room temperature under an Ar atmosphere. 500 mg of the obtained carbon-coated SiMPs were homogeneously dispersed in an ethanol/water (v/v = 1/1, 200 mL) mixture through constant sonication. This suspension was heated to 70°C before 4.0 g NaOH was added. After keeping it at this temperature for a certain time, the mixture was filtered under vacuum followed by washing three times with deionized water and ethanol and then dried at 80°C for 12 h to obtain the final SiMP@C powder. The degree of etching of the carbon-coated SiMPs can be tuned by controlling the etching time. In order to determine the appropriate etching condition, electrodes of SiMP@C with different etching degrees were prepared and assembled into coin-type half cells for galvanostatic charge and discharge measurements. Considering the trade-off between capacity and cycling stability, SiMP@C with an 83% Si content was selected and the Si content in the corresponding SiMP@C-GN reached 66% (Fig. S25). Notably, NaOH etching has almost no influence on the intrinsic structure of SiMPs and CVD carbon cages (Fig. S26). The electrochemical behaviors in first discharging process between SiMP@C before and after NaOH etching are almost the same (Fig. S27).

**2. Synthesis of SiMP@C-GN**

300 mg of SiMP@C powder was firstly dispersed in 50 mL ethanol under sonication to form suspension A. Then, 170 mg of GO powder, prepared through the well-known modified Hummers method, was dispersed in 35 mL deionized water under sonication to form suspension B. Afterwards, suspensions A and B were mixed to form a homogeneous solution to experience hydrothermal reaction at a temperature of 180°C for 6 h, obtaining a cylindrical hybrid hydrogel. Finally, the capillary drying technology was employed to shrink this hydrogel into a dense monolith (~20× volume contraction), yielding the SiMP@C-GN product. Specifically, the hydrogel was subjected to an evaporation-induced drying at 70°C for 48h under air and atmospheric pressure. Notably, the capillary shrinkage process driven by the surface tension of the trapped water allowed the crumple and densified graphene network tightly adhered to the surface of SiMP@C to form a 3C architecture.

**3. Electrode fabrication**

The 10 mL of deionized water was first mixed with 100 mg aniline monomer and 200 mg phytic acid to form solution A. 300 mg ammonium persulfate was added to another 10 mL of deionized water to form solution B. Subsequently, 60 mg of each active material (SiMP@C-GN, SiMP@C, and SiMP) was mixed with 0.18 mL solution A and 0.06 mL solution B, and the mixture was subjected to 3 min bath sonication under stirring. The resulting slurry was cast onto a 10 μm-thick copper (Cu) foil and dried at room temperature for 24 h. After drying, the electrode was mechanically calendered and completely washed in deionized water several times to remove excessive phytic acid and dried again in a vacuum at room temperature to obtain the final working electrode.

The anode composes of SiMP-based active materials and binders. The binder content is ~5.8 wt% (Fig. S28). The electrode density is ~1.0 g cm^–3^. The total mass loadings (including active materials and binders) are ~1.0 mg cm^–2^ (~1.5 mAh cm^–2^) for relatively thin electrodes and ~2.3 mg cm^–2^ (~3 mAh cm^–2^) for thick electrodes, respectively. (for the half-cell test), respectively. Apart from the minor capacity contribution of PANi binders and carbon components (Fig. S29), a high Si utilization can be achieved in electrodes.

**4. Cell assembly and electrochemical measurements**

The samples were cut into disks with a diameter of 10 mm and assembled into CR2032 coin-type cells in an argon-filled glove box. For the half-cell test, lithium metal foil was used as the counter electrode and 1 M lithium hexafluorophosphate (LiPF_6_) in 89 vol% ethylene carbonate/diethyl carbonate (EC/DEC) (1:1, v/v) with 10 vol% fluoroethylene carbonate (FEC) and 1 vol% vinylene carbonate (VC) was used as the electrolyte.

For the full cell test, the SiMP@C-GN was paired with a LCO or a NCM811 cathode with an N/P ratio of ~1.1. Before the assembly of a full cell, an electrochemical pre-lithiation process is used for SiMP@C-GN anodes by discharging to 0.1 V with a current density of 0.02 C for two cycles, with a lithium foil pairing as the counter electrode. The NCM811 cathode was prepared by mixing with super P carbon black and polyvinylidene fluoride (PVDF) binder in a mass ratio of 95:2.5:2.5 and casting the slurries on aluminum (Al) foil. The mass loading of the cathode is 12–18 mg cm^–2^ with an areal capacity of 2–3 mAh cm^–2^. Especially, the sole electrochemical performance of NCM811 in a full-cell test (with graphite anodes, Fig. S30) and its electrode stability are also provided in Fig. S31.

The battery performance was evaluated by galvanostatic charge/discharge measurements conducted on a LAND testing system at room temperature with 0.01–1 V for the half cell and 2.5–4.2 V for the full cell versus Li^+^/Li. Electrochemical impedance spectroscopy was performed with an alternating current voltage amplitude of 5 mV in the frequency range 100 kHz to 0.01 Hz using an electrochemical workstation (Metrohm Autolab, Switzerland).

**5. *In situ* TEM characterization**

*In situ* TEM experiments were performed on a JEOL-3100 FEF, which is equipped with a Nanofactory Instruments STM-TEM holder. The electrochemical micro-cell was assembled with an Au rod used as the working electrode and a small piece of Li covered with a layer of Li_2_O attached to the tip of a W probe as the counter electrode. The active materials SiMP@C or SiMP@C-GN were decorated on the edge of the Au electrode, which was attached to a piezo-manipulator. Before the *in situ* TEM observations, the active SiMP@C or SiMP@C-GN was loaded onto the edge of an Au rod with a freshly cut tip by simply scratching the Au rod against bulk powder of the SiMP@C or SiMP@C-GN. *In situ* lithiation was carried out at a negative bias of –2 V with respect to the Li metal.


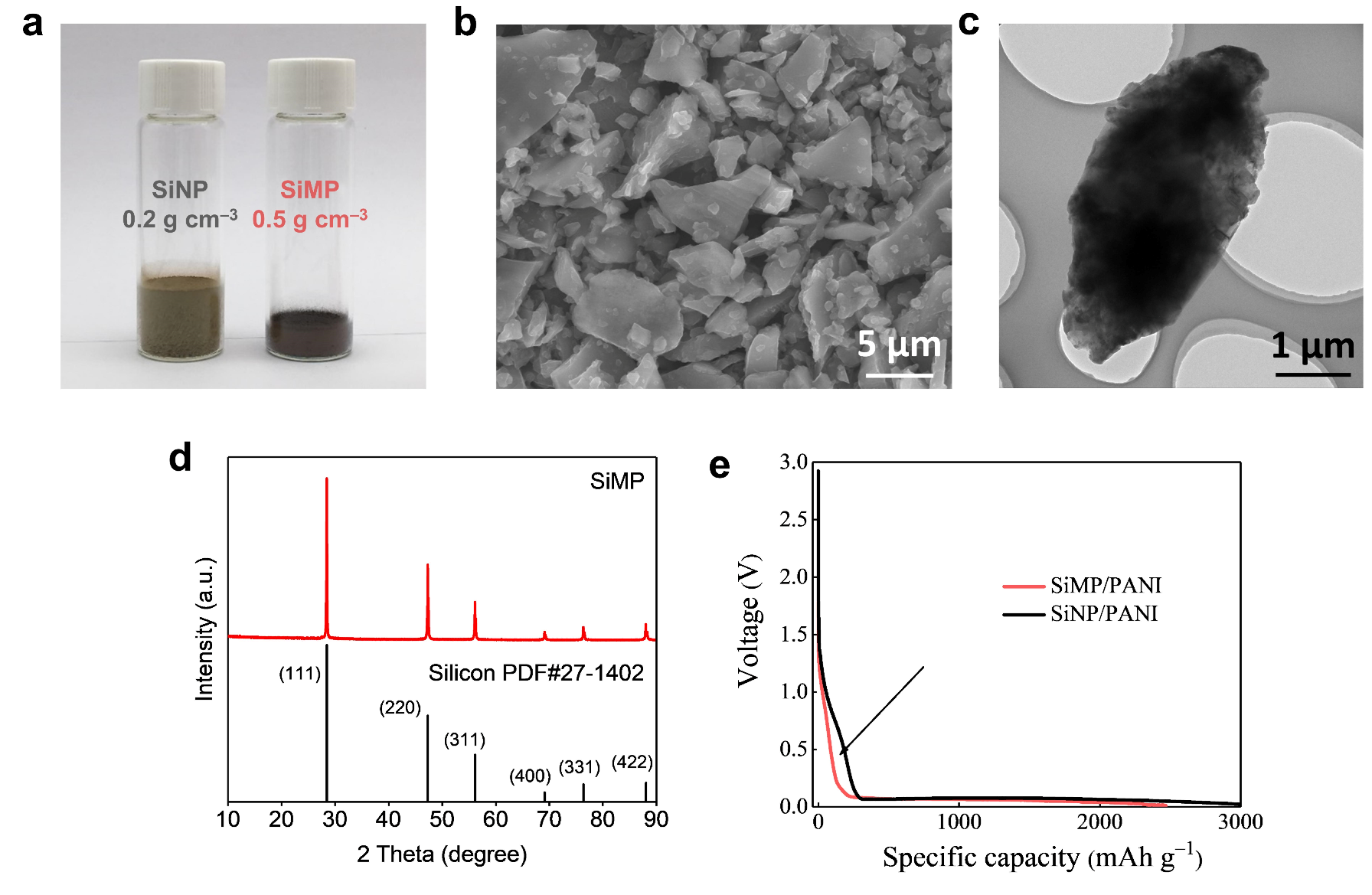


**Fig. S1. Characterization of bare silicon microparticles.** (**a**) Digital image of bare Si nanoparticles (left) and microparticles (right), showing that the tap density of SiMP is much larger than SiNP. (**b**) SEM image of bare SiMP, noting that it does not have regular shape. (**c**) TEM image of bare SiMP. (**d**) XRD spectrum of SiMP. (**e**) The first-cycle discharge curve of SiMP and SiNP anodes.

**
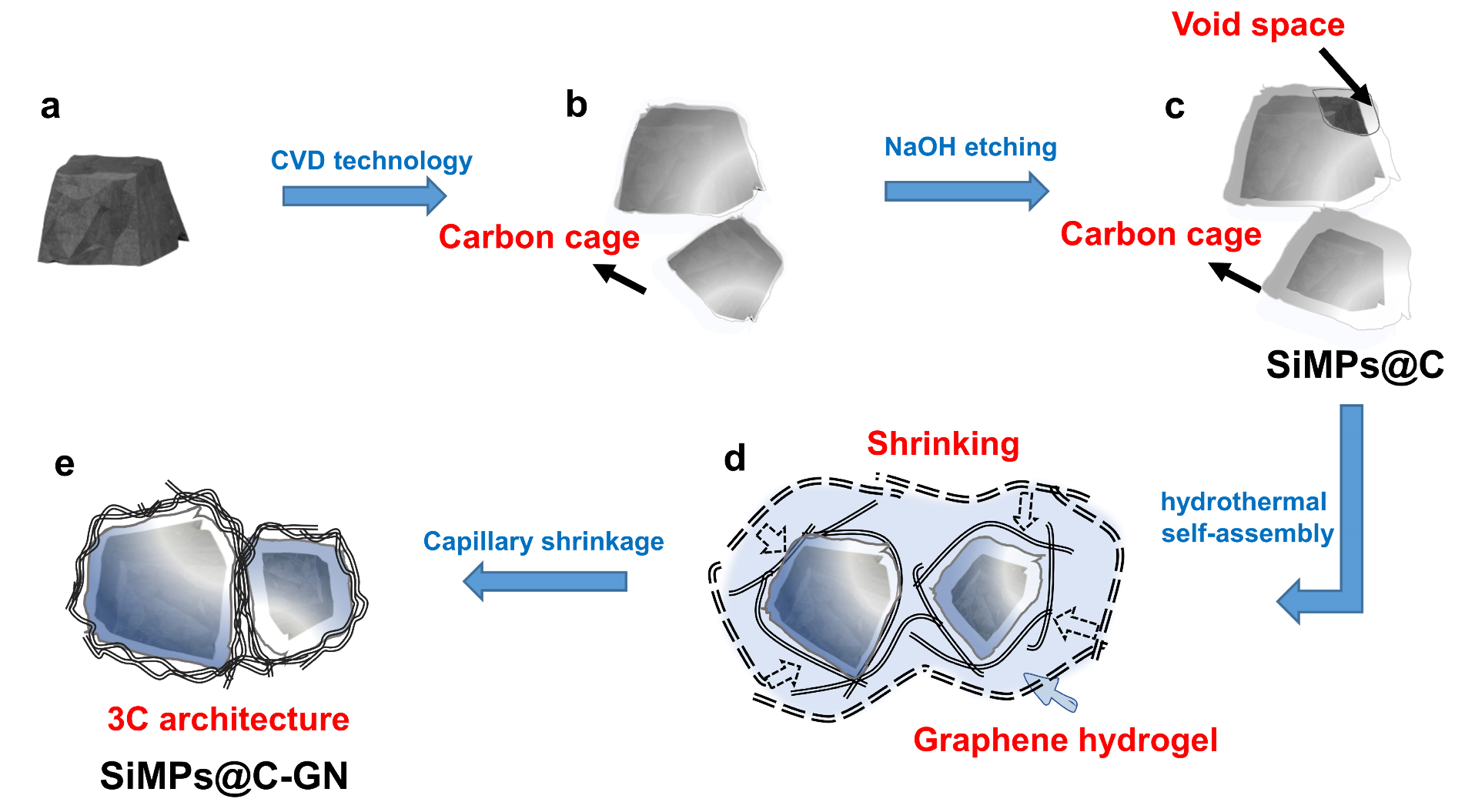
**

**Fig. S2. Schematic of 3C architecture fabrications.**

**
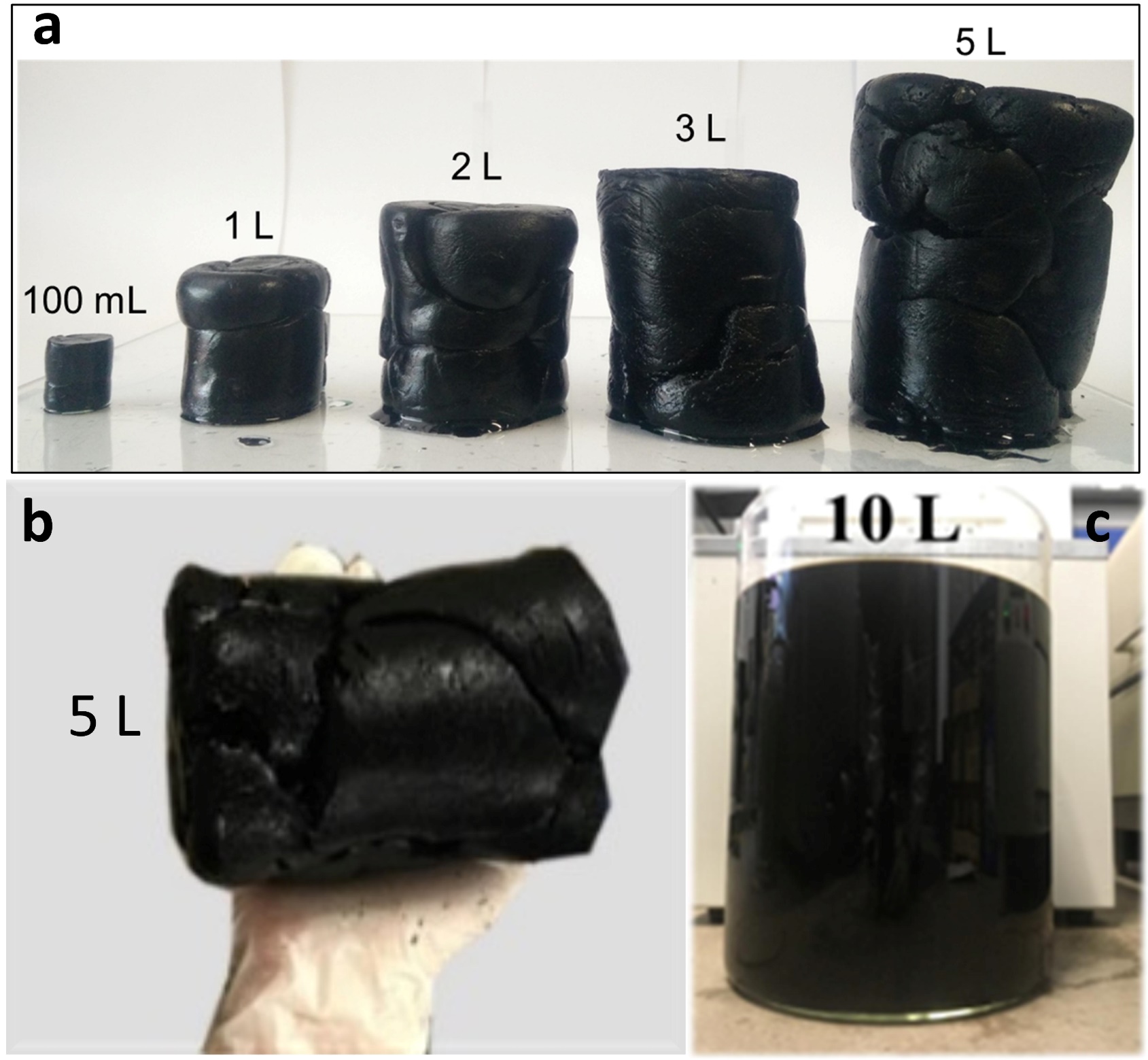
**

**Fig. S3. Scalable liquid-assembly of GO for encapsulating SiMPs.** (**a**) Increased size of graphene hydrogel with increased GO usage. (**b**) The size of graphene hydrogel produced by using 5 L GO compared with a human hand. (**c**) A 10 L container for scalable liquid assembly of GO, which can produce a dense SiMP@C-GN materials at a kg scale.





**Fig. S4. Thermogravimetric analysis (TGA) of SiMP@Cs with different silicon content obtained by controlling the etching time.**

**
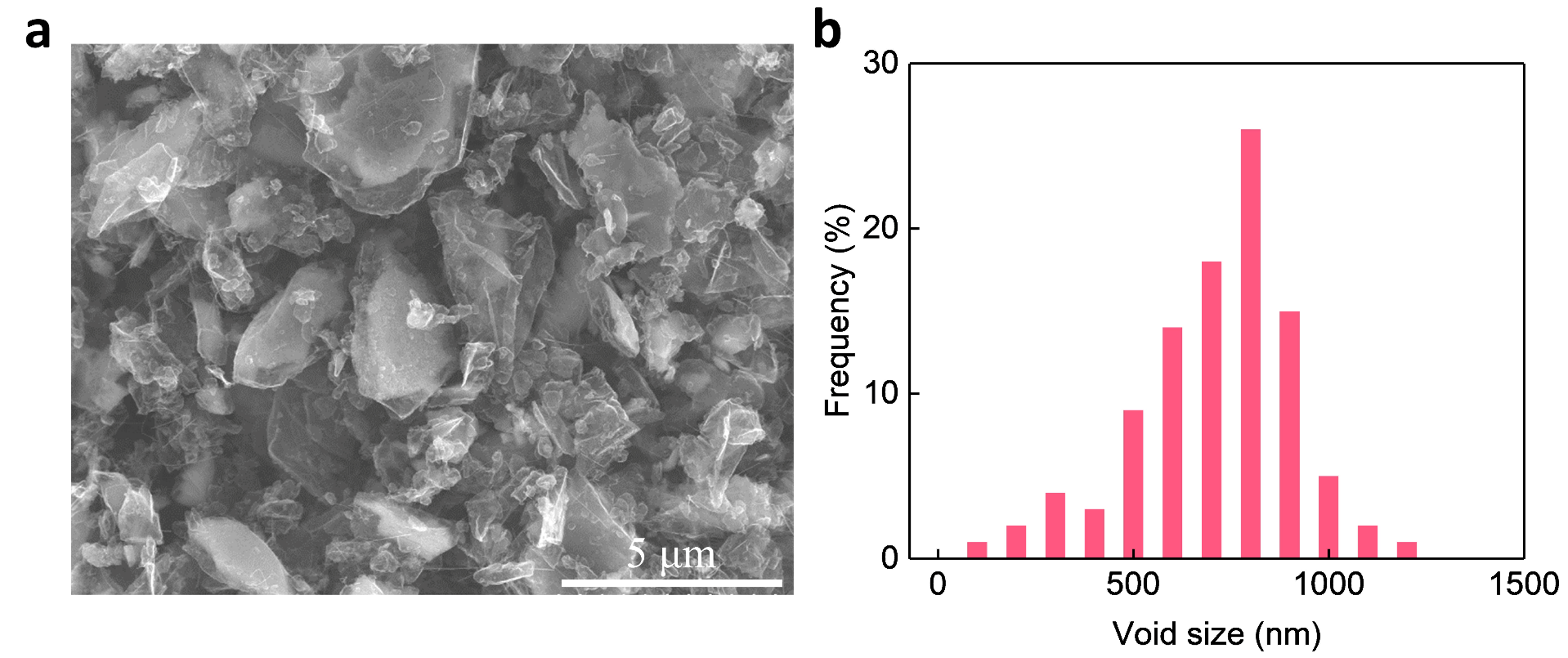
**

**Fig. S5.** **Characterization of etched SiMP@C particles.** (**a**) A broad-view SEM image of SiMP@C. (**b**) Distribution of etched space of 100 SiMP@C particles.

**
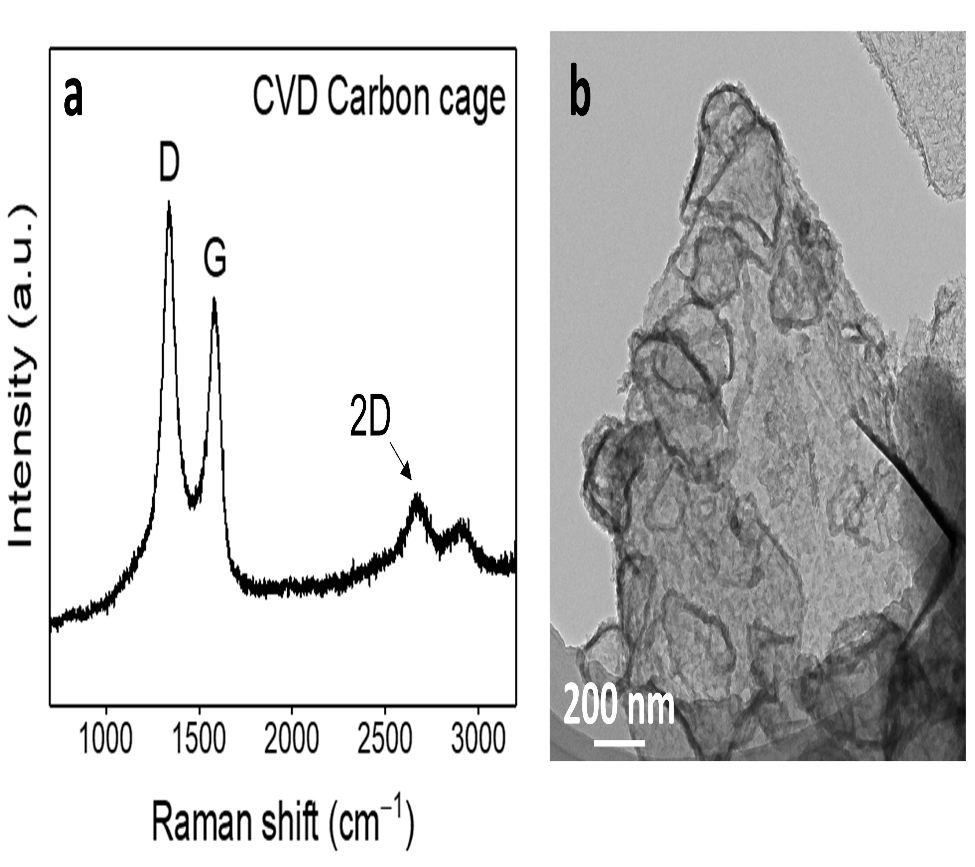
**

**Fig. S6.** **Characterization of CVD carbon cages.** (**a**) Raman spectrum of CVD carbon cages. (**b**) TEM image of CVD carbons.

**

**

**Fig. S7. The morphology of SiMP@C-GN hydrogel (left) and dense monolith (right) after employing a typical capillary drying technique.**

**
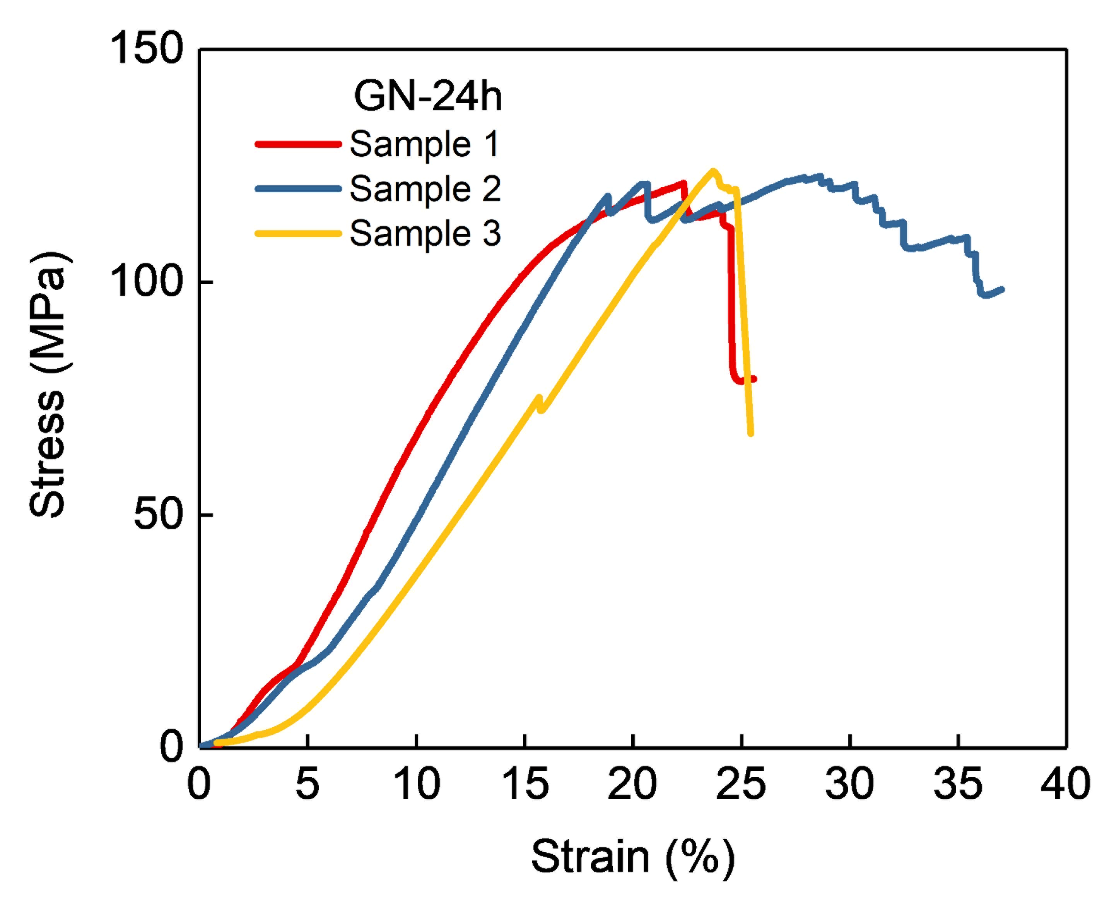
**

**Fig. S8. The reproducibility of stress-strain curves of three samples of GN-24h.**


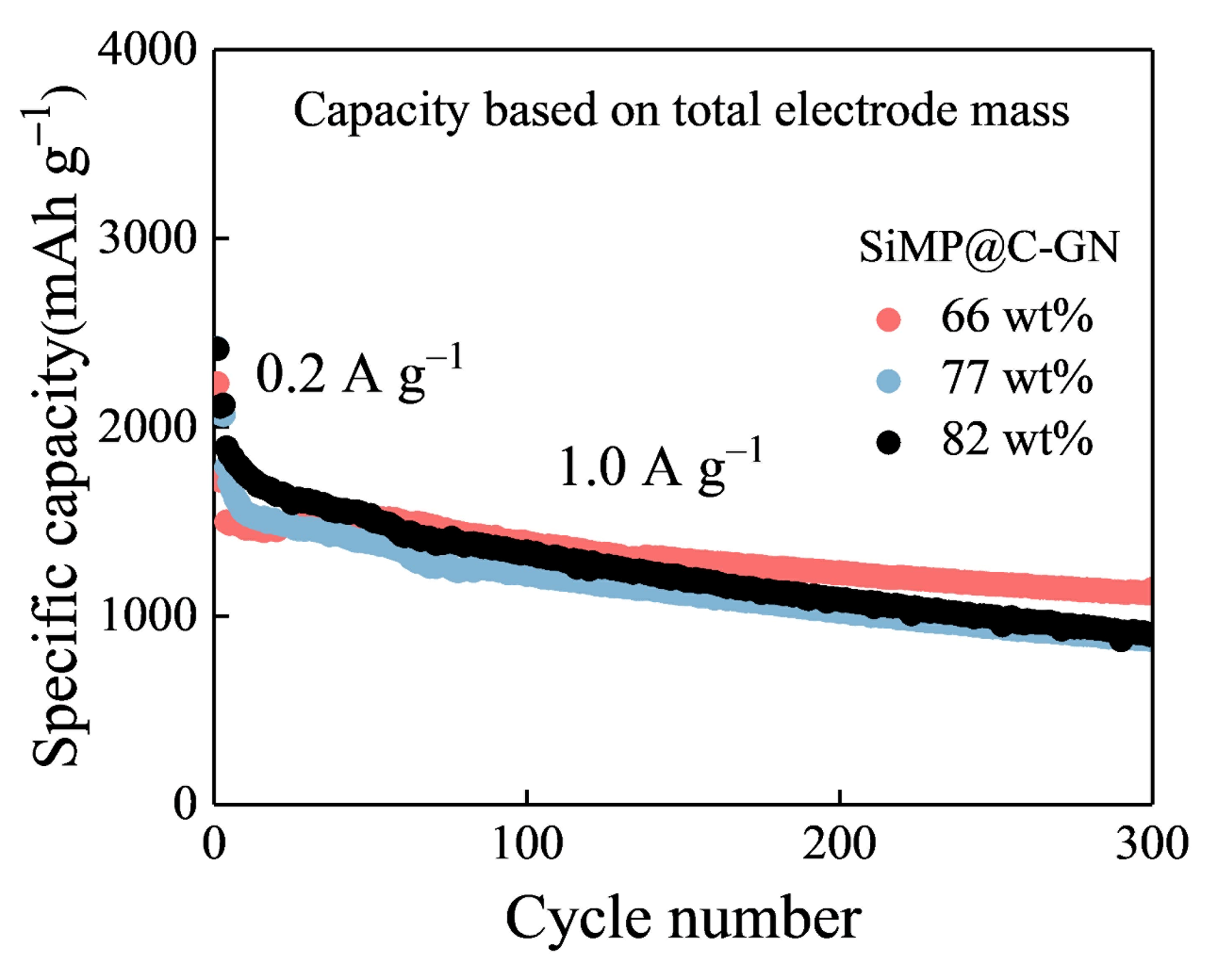


**Fig. S9. The cycling performance of SiMP@C-GN with different Si contents, also with different graphene amounts.**


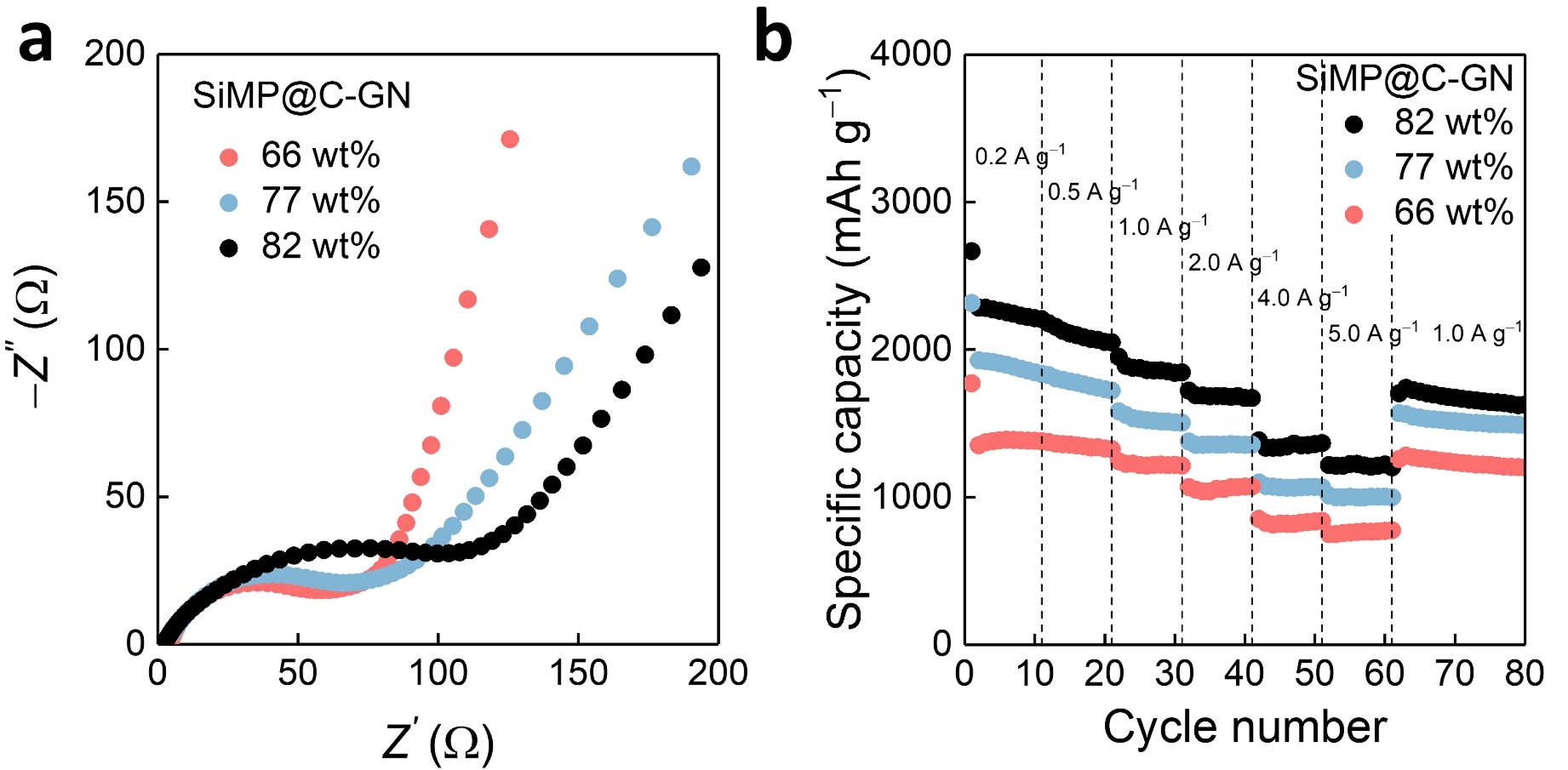


**Fig. S10.** **Electrochemical characterization of SiMP@C-GN with different Si mass contents.** (**a**) EIS. (**b**) Rate performance.


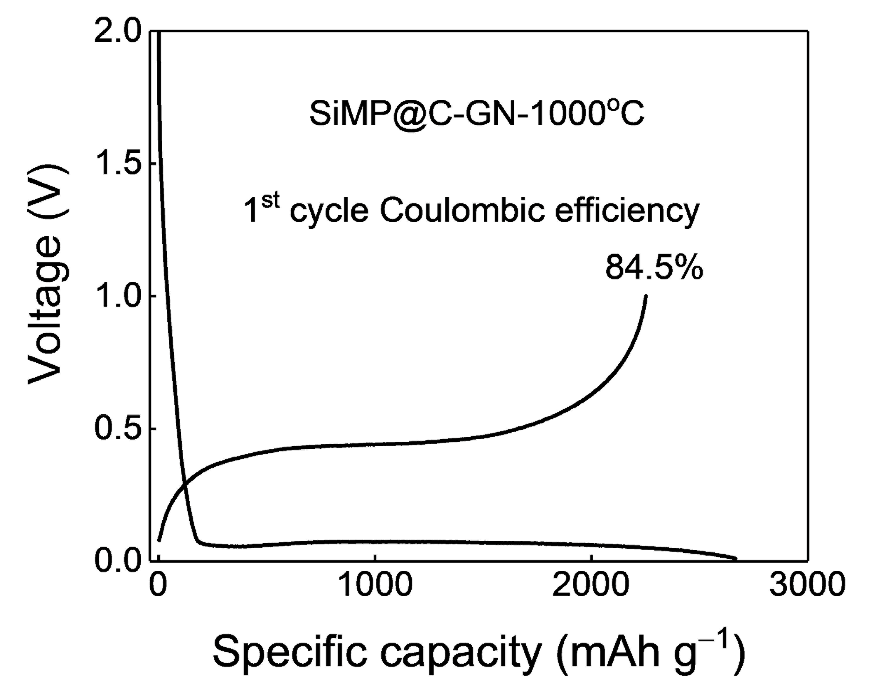


**Fig. S11. The optimized initial Columbic efficiency of SiMP@C-GN with 1000 ºC thermal treatment.**


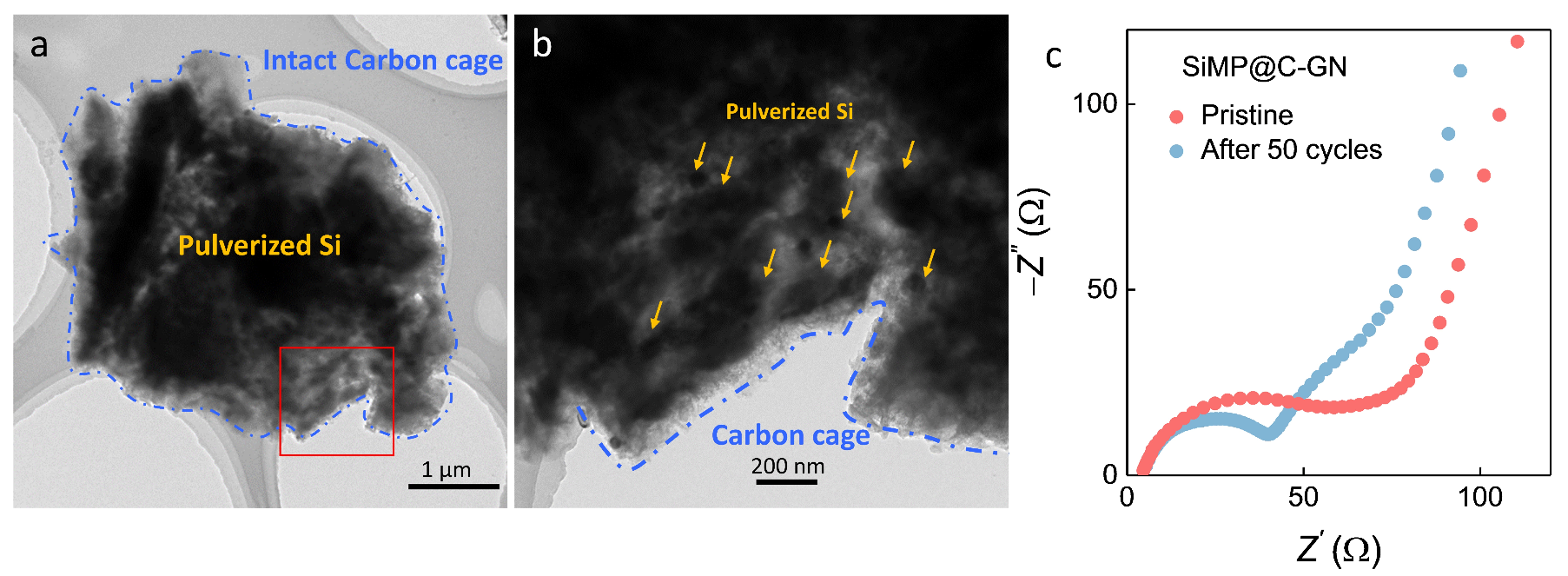


**Fig. S12. Characterization of SiMP@C-GN after cycling.** (**a**) TEM image of SiMP@C-GN after cycling (50 cycles). (**b**) Enlarged view of the boxed area in (**a**). (**c**) EIS of SiMP@C-GN before and after cycling (50 cycles).





**Fig. S13. Characterization of the integrity of carbon cage after cycling.** (**a**) Higher-magnification TEM image and (**b-d**) EDS mapping of SiMP@C-GN after cycling showing the distribution of elemental C and Si.


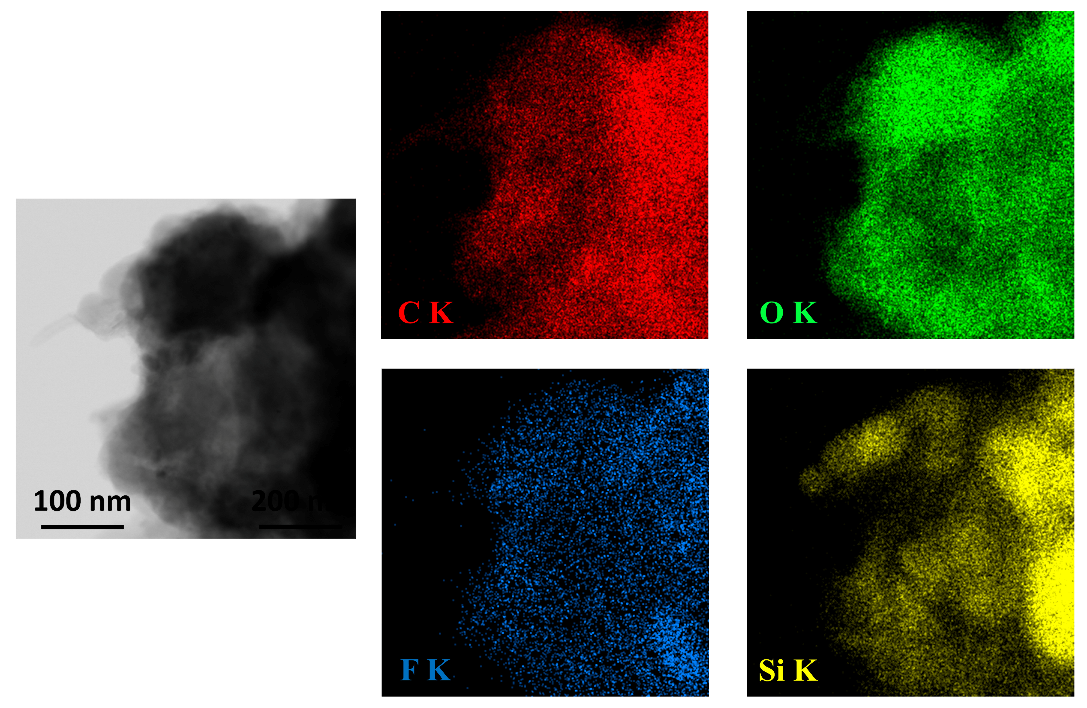


**Fig. S14. TEM-EDS mapping of SiMP@C-GN anodes after 200 cycles.**


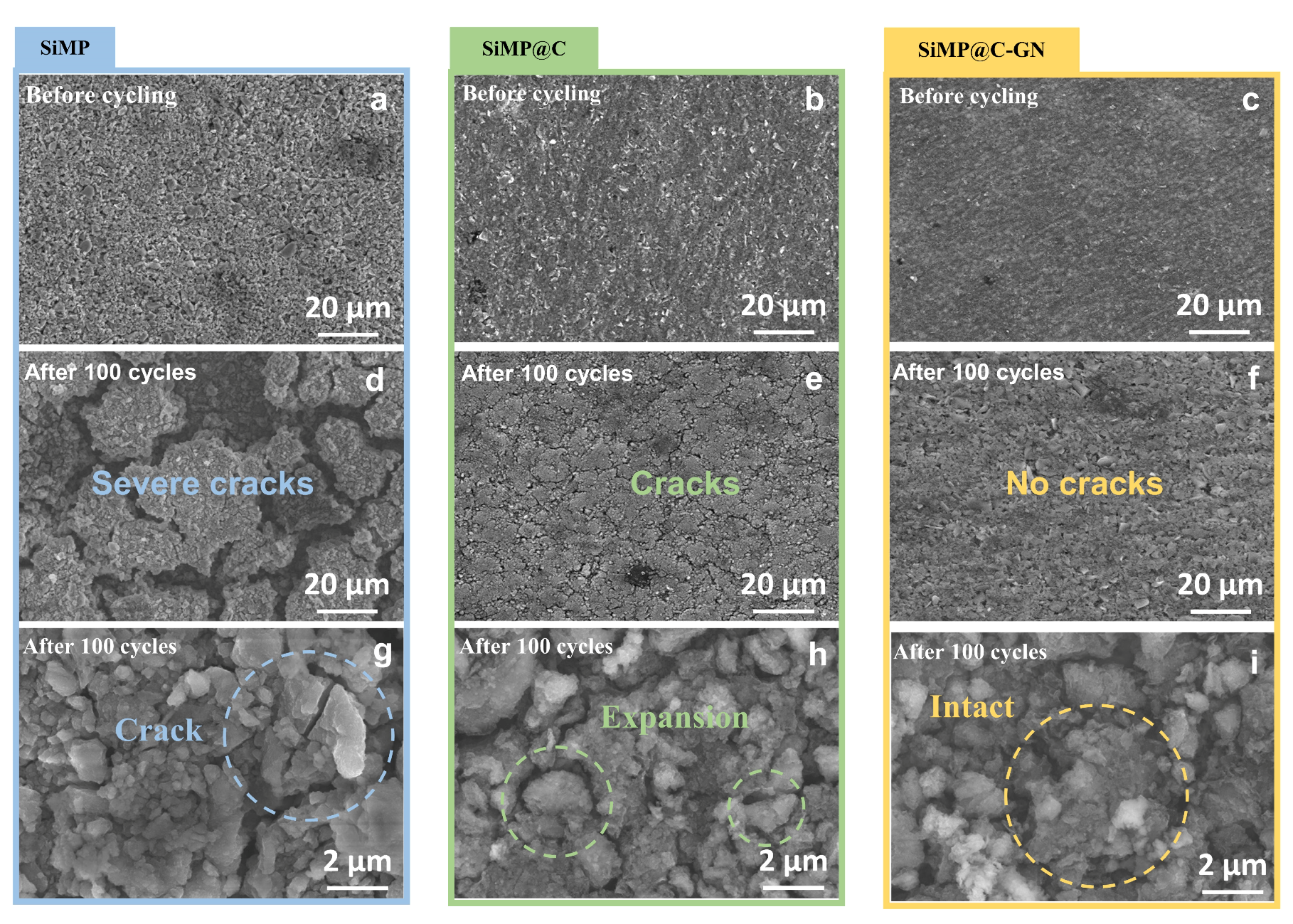


**Fig. S15.** SEM images of (**a**) SiMP, (**b**) SiMP@C and (**c**) SiMP@C-GN electrodes before cycling. SEM images of (**d**, **g**) SiMP, (**e**, **h**) SiMP@C and (**f**, **i**) SiMP@C-GN electrodes after 100 cycles, respectively.

**

**

**Fig. S16. Thermogravimetric analysis of SiMP@C-GN with/without etching and SiMP-GN.**

**
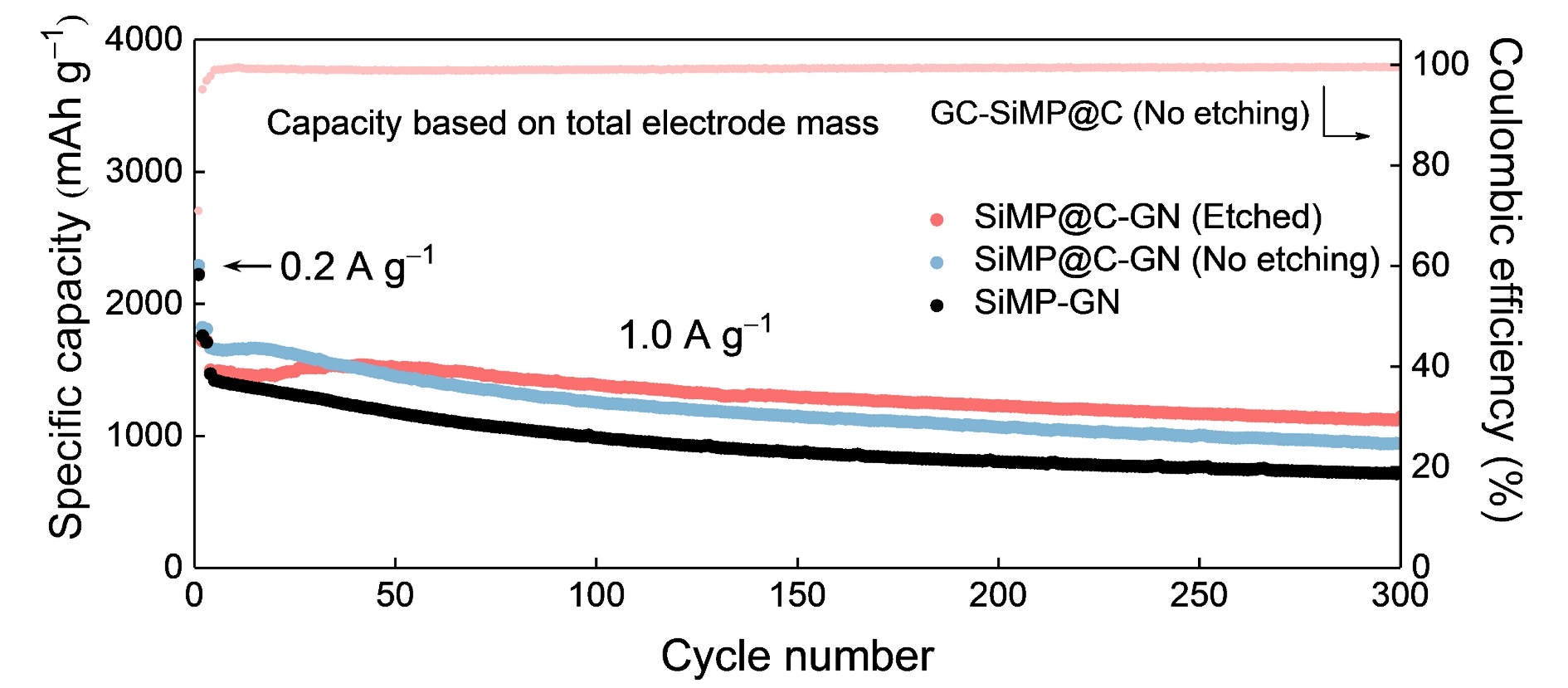
**

**Fig. S17. Half-cell discharge capacities of SiMP@C-GN, SiMP@C-GN without etching and SiMP-GN anodes.** The corresponding Coulombic efficiency of SiMP@C-GN anode without etching is also displayed. The charge/discharge current density is 0.2 A g^–1^ for the initial three cycles and 1.0 A g^–1^ for later cycles.


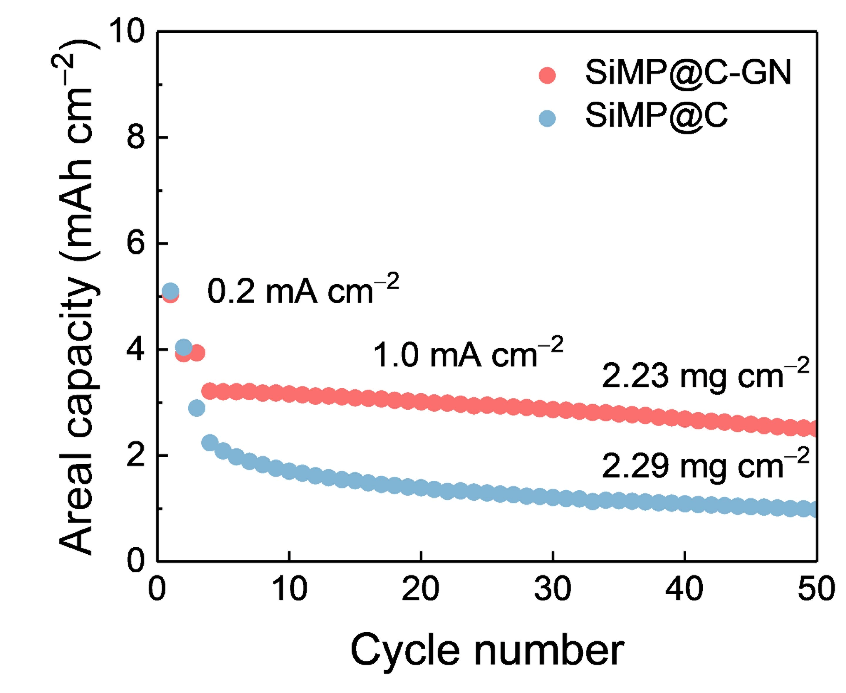


**Fig. S18.** **Cycling performance of high-mass-loaded SiMP@C-GN and SiMP@C anodes (equal initial areal capacity at 0.2 mA cm^–2^).** All electrodes were first cycled at 0.2 mA cm^–2^ for the first three cycles and 1.0 mA cm^–2^ for later cycles.





**Fig. S19. Cross-section SEM images of SiMP@C and SiMP@C-GN electrodes before cycling and after 50 cycles.**


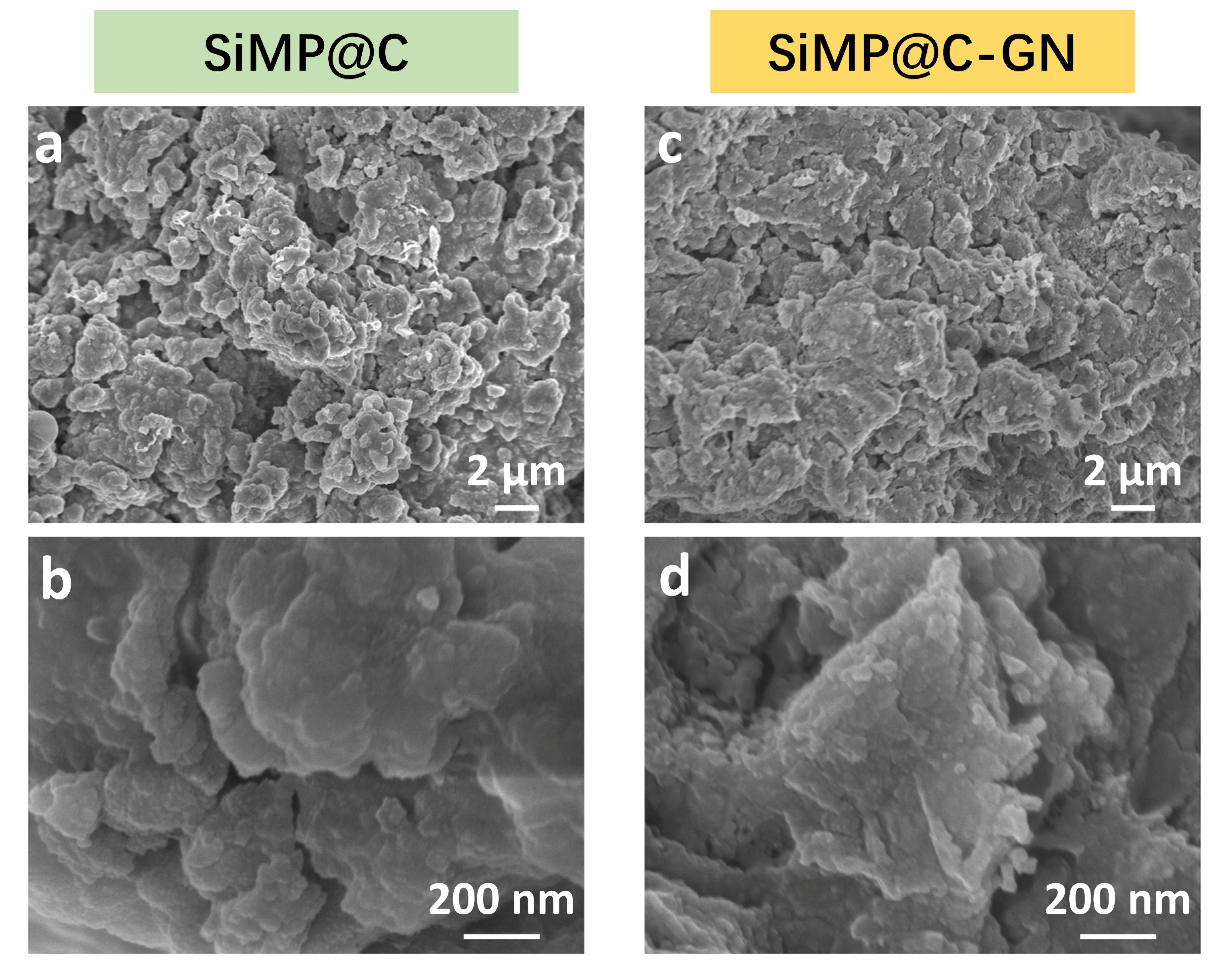


**Fig. S20.** High magnification SEM images of (**a**, **b**) SiMP@C and (**c**, **d**) SiMP@C-GN particles in anodes after 50 cycles.


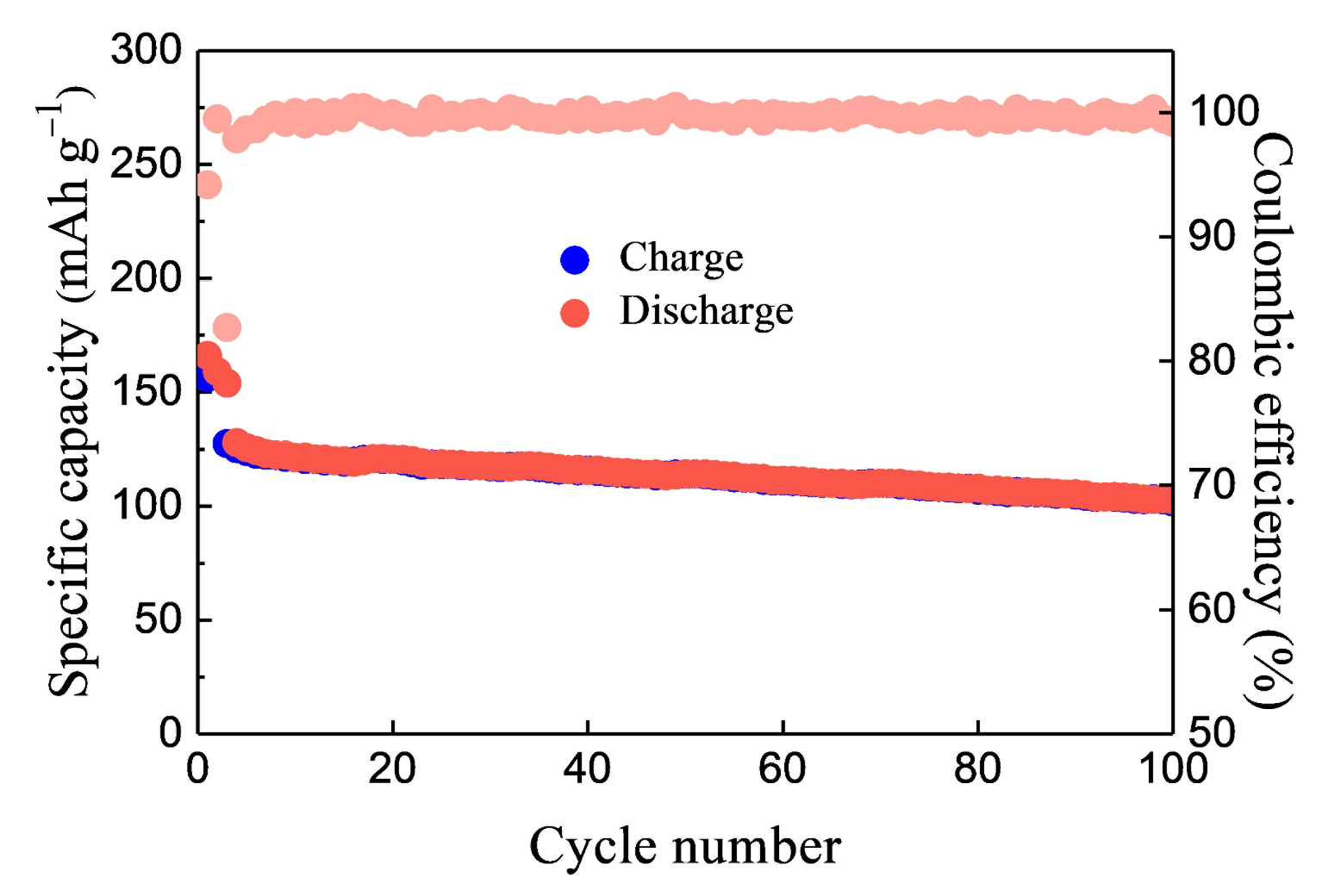


**Fig. S21. Cycling performance and Coulombic efficiency change of the SiMP@C-GN/LCO full cell.** The charge/discharge current density is 0.1C for the first two cycles and 0.5C for later cycles.


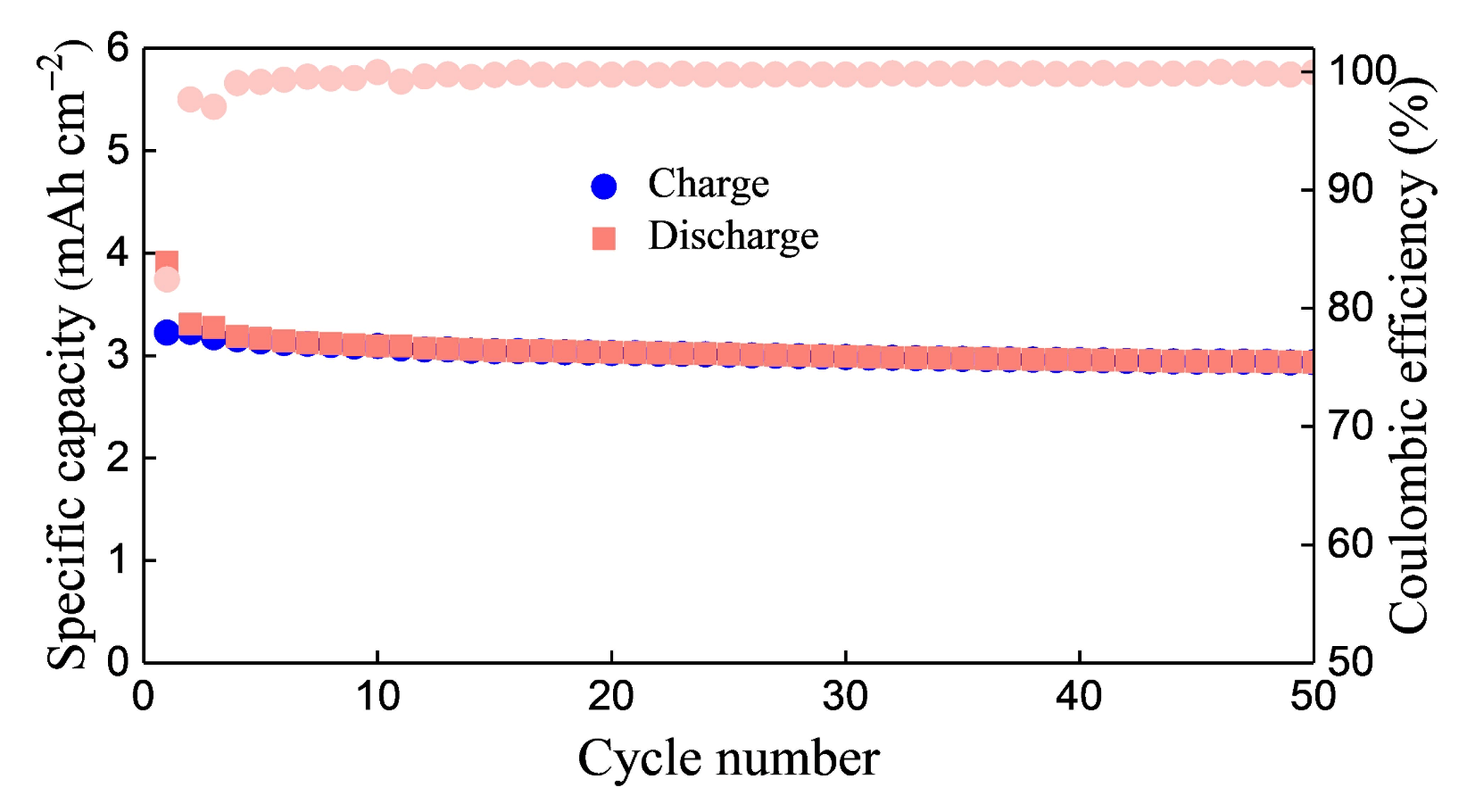


**Fig. S22. Cycling performance and Coulombic efficiency of the SiMP@C-GN/NCM811 full cell.** The charge/discharge current density is 0.1C for the first two cycles and 0.5C for later cycles.


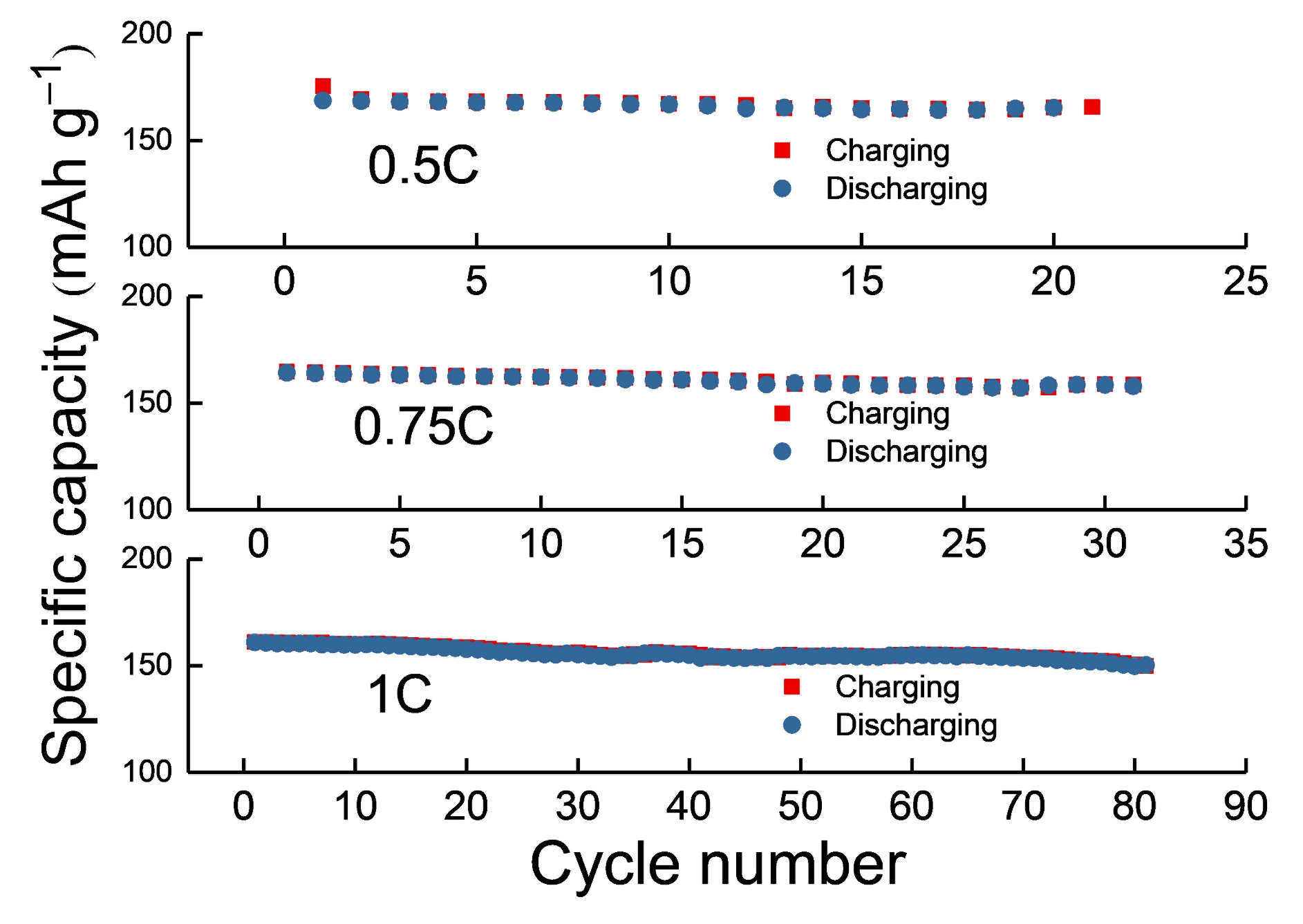


**Fig. S23.** **The cycle stability of SiMP@C-GN evaluated by a full cell system after an activation process under various current densities (0.5C, 0.75C and 1C).**





**Fig. S24.** **Characterization of the pouch cell.** (**a**) Electrolyte usage (3.5 g Ah^-1^) in the 1.2 Ah six-layer pouch cell (5.3 cm×6.7 cm, 35.51 cm^2^). Total weight of the pouch cell is 22.7828 g, and the electrolyte weight is 4.3963 g. (**b**) Photo of the six-layer thick pouch cell. (**c**) Cycling performance of this six-layer full cell. The charge/discharge current density is 0.1C for the first two cycles and 0.5C for later cycles.


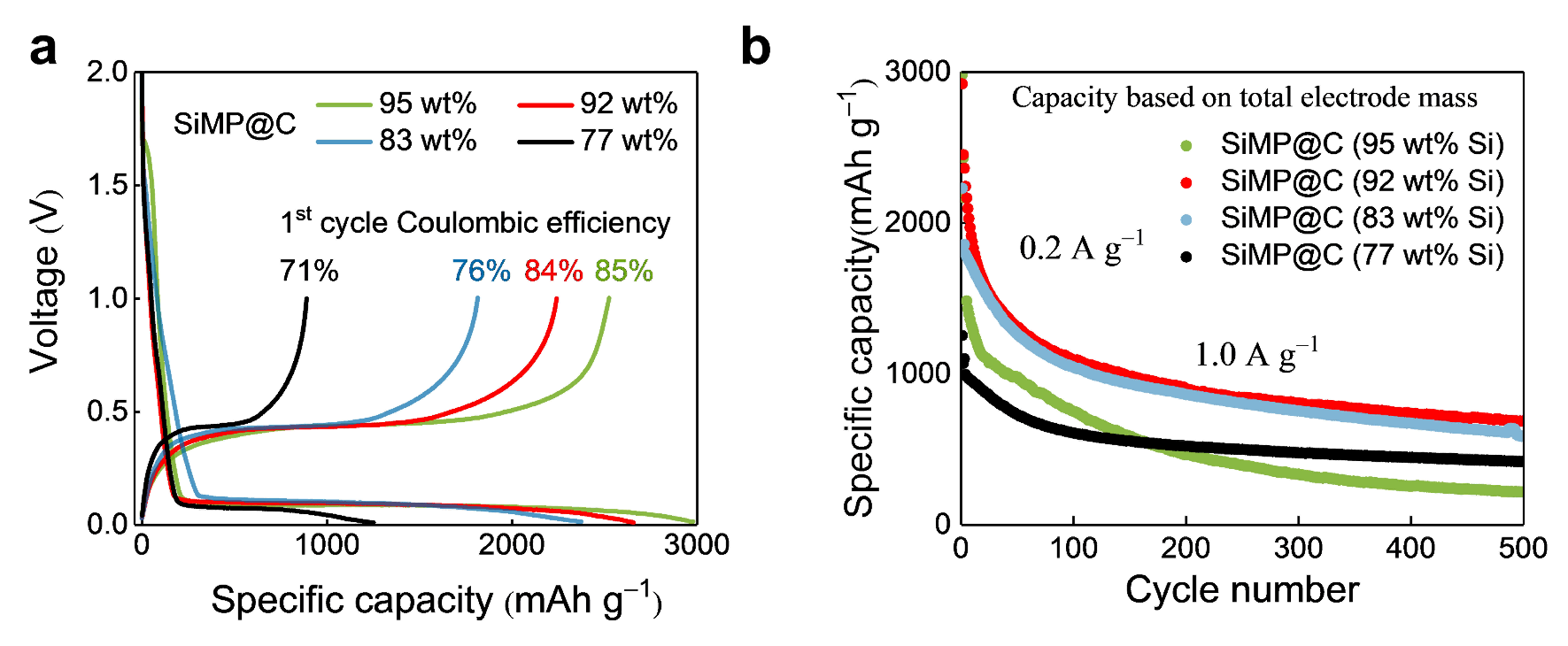


**Fig. S25. Electrochemical characterization of SiMP@C anodes with different silicon content.** (**a**) First-cycle galvanostatic charge/discharge voltage profiles of SiMP@C anodes measured at 0.2 A g^–1^ with corresponding Coulombic efficiencies. (**b**) Half-cell discharge capacities of SiMP@C anodes. The charge/discharge current density is 0.2 A g^–1^ for the initial three cycles and 1.0 A g^–1^ for later cycles.


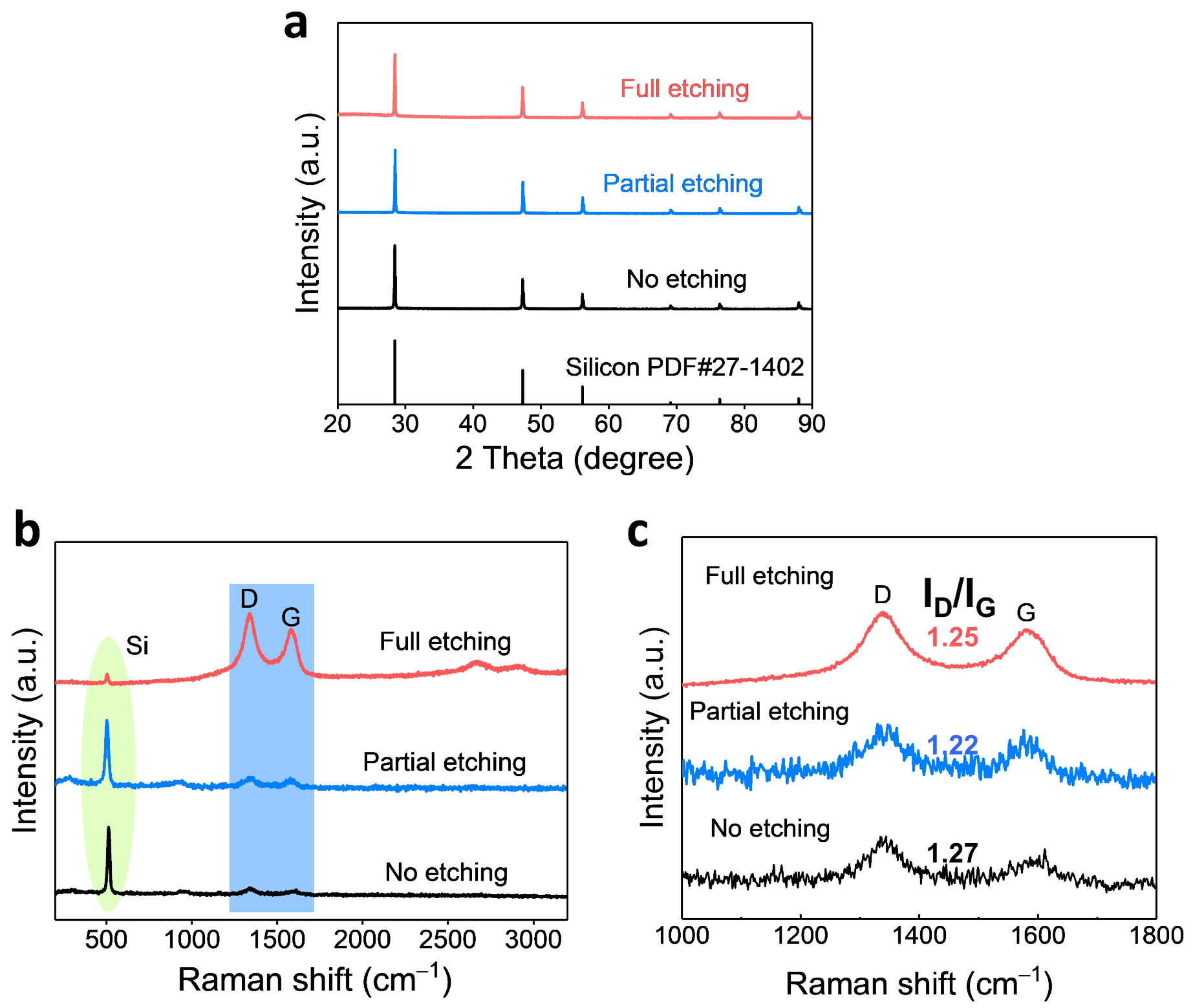


**Fig. S26.** **Characterization of CVD carbons before and after NaOH etching.** (**a**) XRD patterns of SiMP@C before and after NaOH etching. (**b**) Raman spectra of SiMP@C with and without the NaOH etching. (**c**) Normalized Raman peaks of CVD carbons with and without NaOH etching. With the increase of etched Si components, there is almost no change of the Raman peak of CVD carbons indicated by the constant I_D_/I_G_ values ~ 1.25.


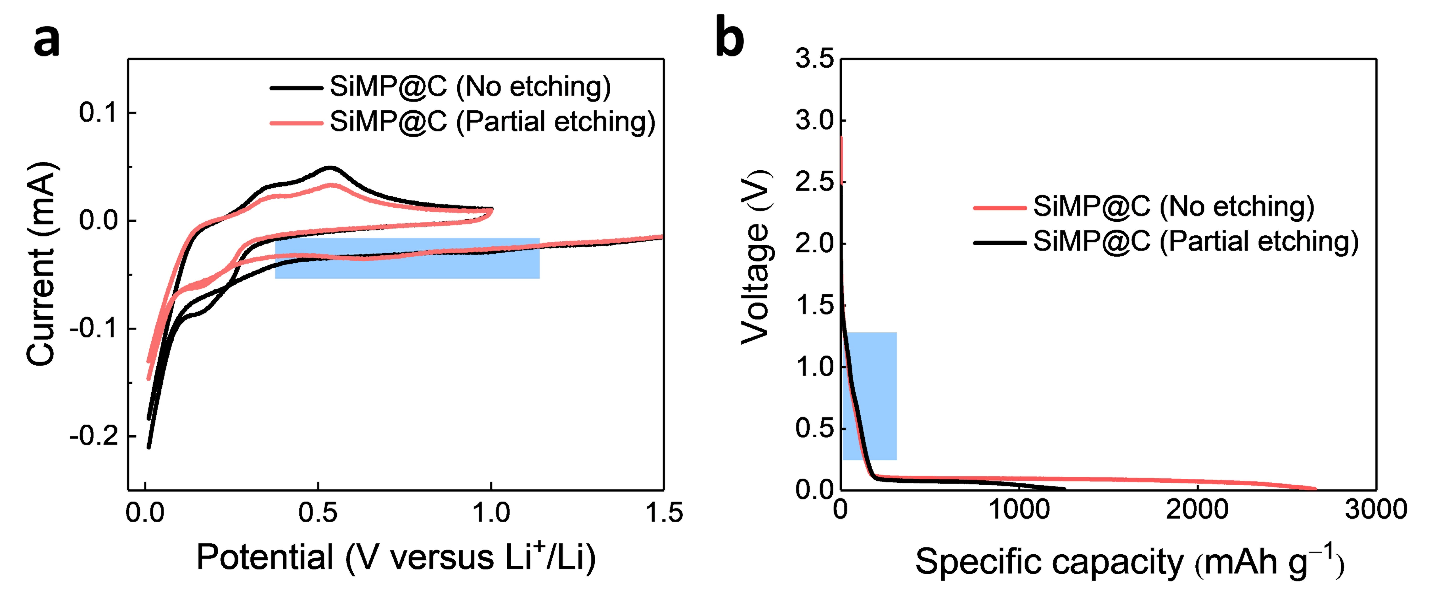


**Fig. S27.** **Electrochemical characterization of SiMP@C with and without NaOH etching.** (**a**) CV curves of SiMP@C with and without NaOH etching. (**b**) The discharge-charge curves of SiMP@Cs with and without NaOH etching.


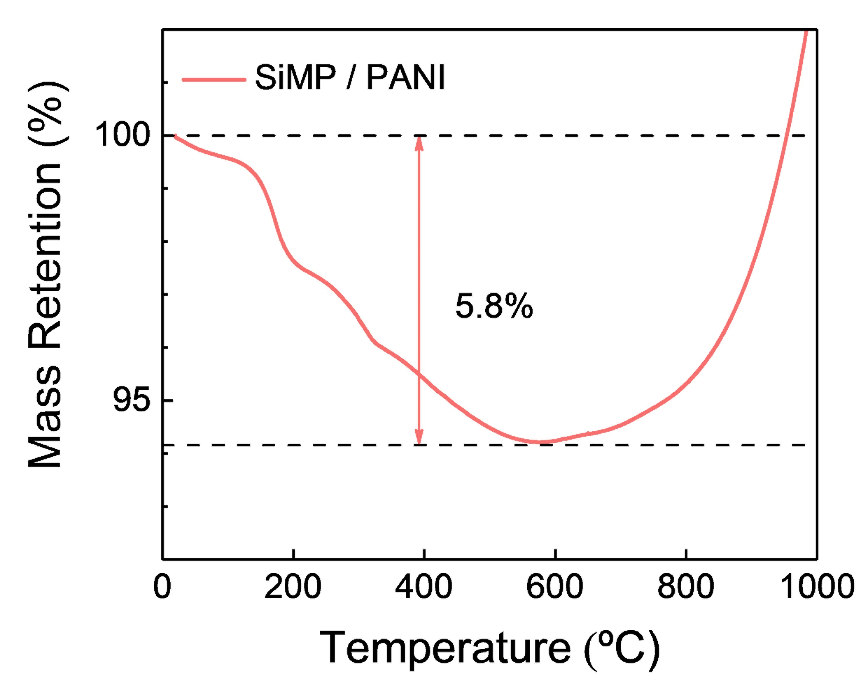


**Fig. S28. TGA curve of the SiMP/PANi electrode material, showing a mass content of PANi binders ~5.8 wt% in the electrode.**


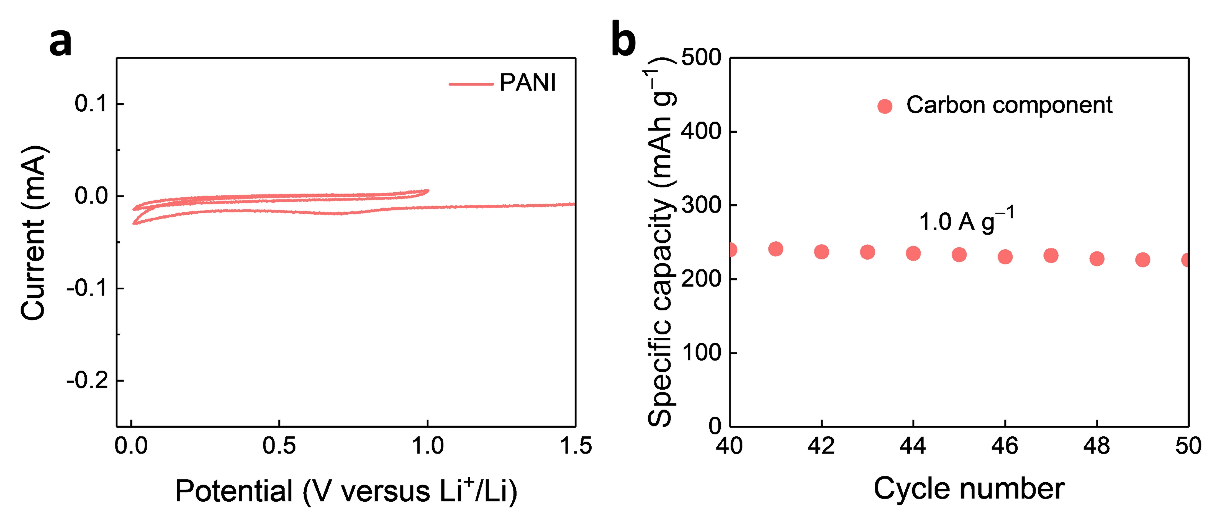


**Fig. S29.** **Capacity contribution of PANi and carbon components**. (**a**) CV curve of PANi. (**b**) Specific capacity of carbon components after a full Si etching.


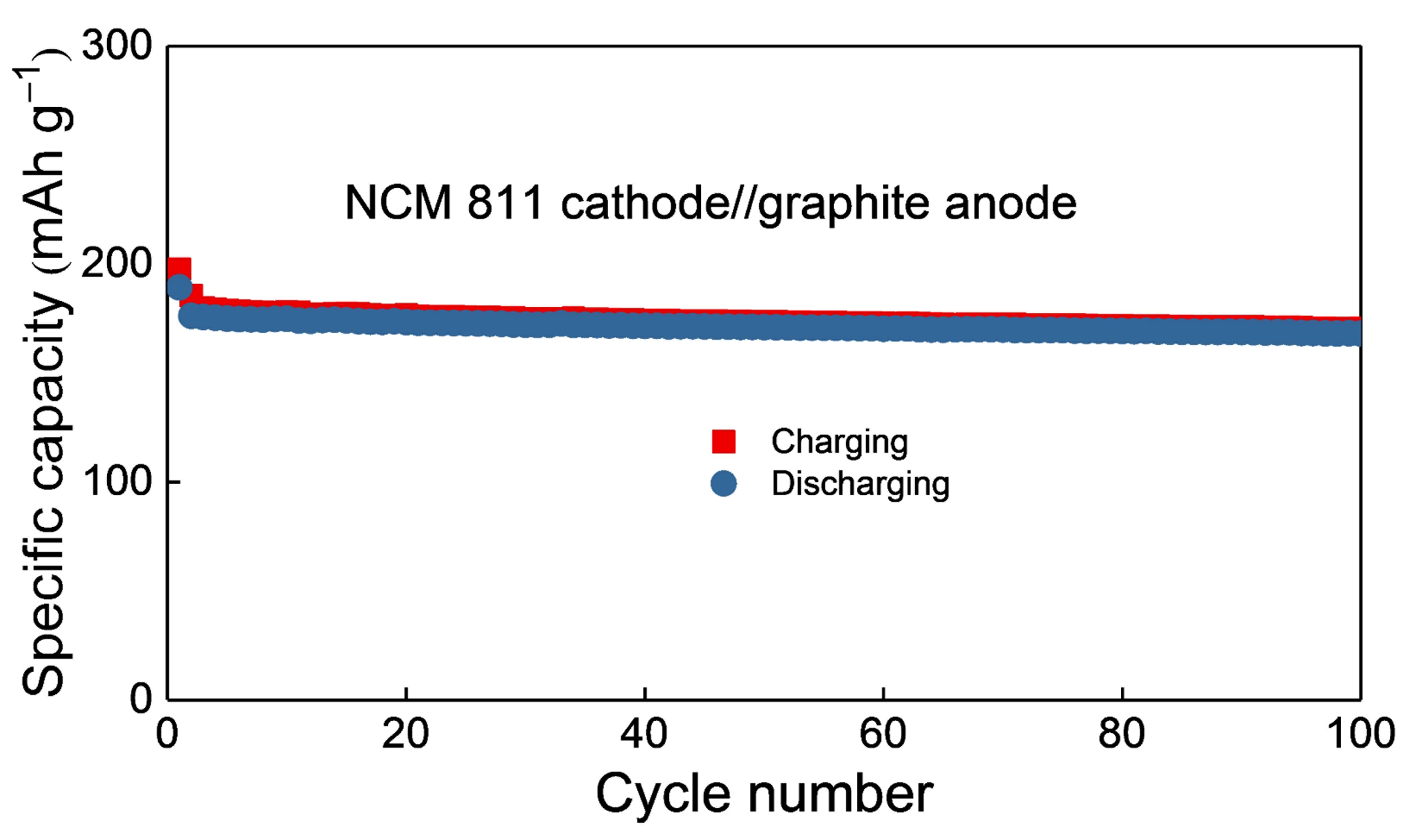


**Fig. S30. Cycling performance of a NCM811//graphite full cell.** The charge/discharge current density is 0.1C for the first two cycles and 0.5C for later cycles.


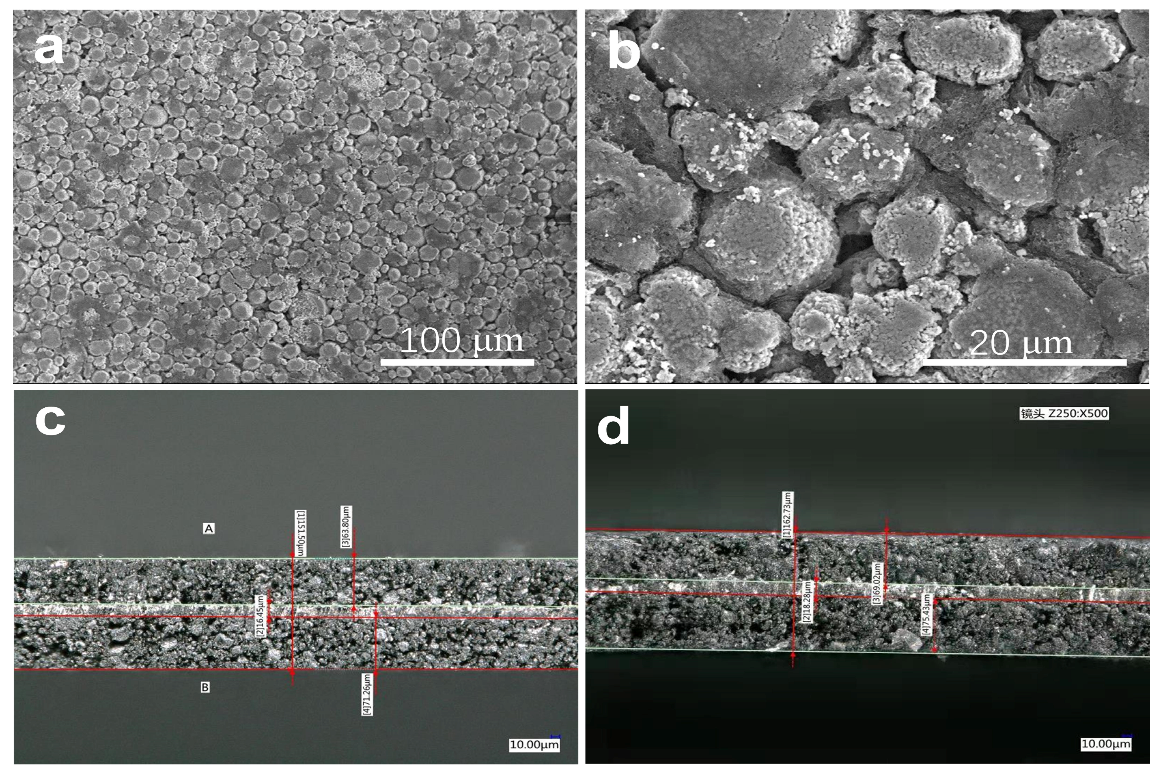


**Fig. S31.** The top-view SEM image of the thick NCM811 cathode in (**a**) a low and (**b**) a relative higher resolution after cycling. The cross-section view SEM image of the thick NCM811 cathode (**c**) before and (**d**) after cycling.

**Table S1.** **The detailed parameters of GN-X (6h, 10h and 24h) cylinders for a compression test.**

| Cylinder sample | | Height（mm） | Diameter（mm） | Ratio of height and diameter | Bottom area（mm^2^） | Force（N） | Strength (MPa) | Strain (%) |
| --- | --- | --- | --- | --- | --- | --- | --- | --- |
| **GN-6h** | 9.1 | | 8.2 | 1.1 | 53.8 | 23 | 0.43 | 21 |
| **GN-10h** | 8.2 | | 6.6 | 1.2 | 34.2 | 33 | 0.96 | 11 |
| **GN-24h-1** | 3.4 | | 3.7 | 0.92 | 10.7 | 1294 | 121 | 22 |
| **GN-24h-2** | 3.1 | | 3.4 | 0.91 | 9.1 | 1107 | 122 | 28 |
| **GN-24h-3** | 3.2 | | 3.6 | 0.89 | 10.2 | 1254 | 124 | 23 |

**Table S2. Comparison with representative Si anodes in a half-cell test.**

| Materials | Cost (high or low) | Gravimetric capacity based on the whole electrode | Density of electrode  (g cm^−3^) | Volumetric capacity based on the electrode (mAh cm^−3^) | Cycles | Current  Density | Voltage | Active material mass loading (mg cm^−2^) | Ref |
| --- | --- | --- | --- | --- | --- | --- | --- | --- | --- |
| **SiMP@C-GN** | **Low**  **(3–5 μm)** | **1717** | **1.0-1.1** | **1717** | **1** | **0.2 A g^−1^** | **0.01–1 V** | **1.0** | **This work** |
|  |  | **1386** |  | **1386** | **100** | **1.0 A g^−1^** |  |  |  |
|  |  | **1141** |  | **1141** | **300** |  |  |  |  |
|  |  | **1030** |  | **1050** | **500** |  |  |  |  |
|  |  | **774** |  | **774** | **1000** |  |  |  |  |
| **PR-PAA-SiMP** | **Low**  **(1–5 μm)** | **~1250**  **~1160** | **~1.3** | **1625**  **1508** | **220**  **400**  **(Li metal change)** | **1.25 mA cm^−2^** | **0.01–1.5 V** | **1.04** | **[1]** |
| **SiMP@Gr** | **Low**  **(1–3 μm)** | **~1400** | **0.6** | **840** | **300** | **2.1 A g^−1^** | **0.01–1 V** | **~0.8** | **[2]** |
| **PAA-P(HEA-co-DMA)-SiMP** | **Low**  **(0.5–3 μm)** | **1915** | **N/A** | **N/A** | **220** | **2.1 A g^−1^** | **0.01–1.2 V** | **~1.0** | **[3]** |
| Si/SHP-PEG750(40) (after volume expansion) | Low  (~0.8 μm) | ~845 | 0.5–0.8 | 676 | 150 | ~1.8 A g^−1^ | 0.01–1 V | 0.5–0.7 | [4] |
| **np-ZnO/SiMP** | **Low**  **(1–3 μm)** | **1500** | **N/A** | **N/A** | **210** | **0.84 A g^−1^** | **0.01–1 V** | **~1.65** | **[5]** |
| **Self-healing SiMP** | **Low**  **(3–8 μm)** | **1046.8** | **N/A** | **N/A** | **90** | **0.4 A g^−1^** | **0.01–1 V** | **0.5–0.7** | **[6]** |
| **mSi@OG@RGO** | **Low**  **(1–5 μm)** | **~1450** | **0.46** | **670** | **150** | **2.0 A g^−1^** | **0.02–1 V** | **1.0–1.5** | **[7]** |
| nC-pSiMP | Low  (~1 μm) | 1192 | 0.55 | 656 | 1000 | 1.05 A g^−1^ | 0.01–1 V | ~0.5 | [8] |
| SiO*_x_*/C-CVD | Medium  (~0.5 μm) | 729 | 0.7–1.0 | 729 | 500 | 0.5 A g^−1^ | 0.01–3 V | 1.5–2.0 | [9] |
| Si-SHP/CB (after volume expansion) | Medium  (~0.8 μm) | 1182 | 1.1 | 1301 | 120 | 0.1 mA cm^−2^ | 0.01–1 V | 1.70 | [10] |
| SiNP-PANi | High  (~100 nm) | ~1600 | 0.674 | 1078 | 1000 | 1.0 A g^−1^ | 0.01–1 V | 0.2–0.3 | [11] |
| Si pomegranate | High  (~80 nm) | ~930 | ~0.4 | 372 | 1000 | 2.1 A g^−1^ | 0.01–1 V | ~0.2 | [12] |
| SG-Si-c-PAN | High  (50–70 nm) | 2764 | 0.85 | ~2350 | 100 | 0.1 A g^−1^ | 0.05–1.5 V | 0.8–1.5 | [13] |
| SiNP-alginate | High  (~27 nm) | 1445 | 0.5 | 723 | 100 | 4.2 A g^−1^ | 0.01–1 V | N/A | [14] |
| Si/C microspheres | High  (~100 nm) | 420 | 1.1 | 462 | 500 | 0.3 A g^−1^ | 0.005–1 V | 4.1 | [15] |
| Si−SiO*_x_*−C | High  (~70 nm) | 621 | N/A | N/A | 200 | 4.2 A g^−1^ | 0.01–1.5 V | 1.0 | [16] |
| CVSS/c-CMC-CA | High  (50–70 nm) | 432 | N/A | N/A | 1000 | 5.0 A g^−1^ | 0.01–2.0 V | 0.6–0.9 | [17] |
| Si-alloy/graphite | High  (50–70 nm) | 650 | 1.7 | 1100 | 60 | N/A | 0.01-0.9 V | 6.5 | [18] |

**Table S3. Comparison with representative Si anodes in a full-cell test.**

| **Anode materials** | | **Cathode materials** | **Areal capacity**  **(mAh cm^-2^)** | **Cycles** | **Average voltage** | **Cell geometry** | **Volumetric energy density (Wh L^-1^)** | **Ref** |
| --- | --- | --- | --- | --- | --- | --- | --- | --- |
| SiMP@C-GN | NCM811 | | 3.2 | 50 | 3.7 | Pouch cell | 1048 | This work |
| Si-alloy/graphite | NMC532 | | 3.7 | 290 | N/A | Pouch cell | N/A | [18] |
| Si-graphene | LCO | | 3.0 | 200 | 3.5 | Cylindrical cell | 972 | [19] |
| μ-Si/CNT | NMC/CNT | | 29 | 2 | N/A | N/A | 1030,  1600 (after calendering) | [20] |

**Table S4. Comparison with representative solid SiMP anodes in a full-cell.**

| **Anode materials** | | **Cathode materials** | **Initial reversible areal capacity**  **(mAh cm^-2^)** | **Cycles** | **Ref** |
| --- | --- | --- | --- | --- | --- |
| SiMP@C-GN | NCM811 | | 2.2 | 200 | This work |
| PR-PAA-SiMP | NCA | | 2.4 | 50 | [1] |
| SiMP@Gr | LCO | | 2.2 | 115 | [2] |
| PAA-P(HEA-co-DMA)-SiMP | NCM | | 1.7 | 120 | [3] |

**References**

1 Choi, S. *et al.* Highly elastic binders integrating polyrotaxanes for silicon microparticle anodes in lithium ion batteries. *Science* **357**, 279 (2017).

2 Li, Y. *et al.* Growth of conformal graphene cages on micrometre-sized silicon particles as stable battery anodes. *Nat. Energy* **1**, 15029 (2016).

3 Xu, Z. *et al.* Silicon microparticle anodes with self-healing multiple network binder. *Joule* **2**, 950-961 (2018).

4 Munaoka, T. *et al.* Ionically conductive self-healing binder for low cost Si microparticles anodes in li-ion batteries. *Adv. Energy Mater.* **8**, 1703138 (2018).

5 Kim, D. *et al.* Conversion reaction of nanoporous ZnO for stable electrochemical cycling of binderless Si microparticle composite anode. *ACS Nano* **12**, 10903-10913 (2018).

6 Wang, C. *et al.* Self-healing chemistry enables the stable operation of silicon microparticle anodes for high-energy lithium-ion batteries. *Nat. Chem.* **5**, 1042 (2013).

7 Zhang, X. *et al.* Scallop-inspired shell engineering of microparticles for stable and high volumetric capacity battery anodes. *Small* **14**, 1800752 (2018).

8 Lu, Z. *et al.* Nonfilling carbon coating of porous silicon micrometer-sized particles for high-performance lithium battery anodes. *ACS Nano* **9**, 2540-2547 (2015).

9 Liu, Z. *et al.* Yolk@shell SiO*_x_*/C microspheres with semi-graphitic carbon coating on the exterior and interior surfaces for durable lithium storage. *Energy Storage Mater.* **19**, 299-305 (2019).

10 Chen, Z. *et al.* High-areal-capacity silicon electrodes with low-cost silicon particles based on spatial control of self-healing binder. *Adv. Energy Mater.* **5**, 1401826 (2015).

11 Wu, H. *et al.* Stable Li-ion battery anodes by in-situ polymerization of conducting hydrogel to conformally coat silicon nanoparticles. *Nat. Commun.* **4**, 1943 (2013).

12 Liu, N. *et al.* A pomegranate-inspired nanoscale design for large-volume-change lithium battery anodes. *Nat. Nanotechnol.* **9**, 187 (2014).

13 Hassan, F. M. *et al.* Evidence of covalent synergy in silicon–sulfur–graphene yielding highly efficient and long-life lithium-ion batteries. *Nat. Commun.* **6**, 8597 (2015).

14 Kovalenko, I. *et al.* A major constituent of brown algae for use in high-capacity li-ion batteries. *Science* **334**, 75 (2011).

15 Xu, Q. *et al.* Watermelon-inspired Si/C microspheres with hierarchical buffer structures for densely compacted lithium-ion battery anodes. *Adv. Energy Mater.* **7**, 1601481 (2017).

16 Lee, S. J. *et al.* Delicate structural control of Si–SiO*_x_*–C composite via high-speed spray pyrolysis for Li-ion battery anodes. *Nano Lett.* **17**, 1870-1876 (2017).

17 Liu, Y. *et al.* An all-integrated anode via interlinked chemical bonding between double-shelled–yolk-structured silicon and binder for lithium-ion batteries. *Adv. Mater.* **29**, 1703028 (2017).

18 Marinaro, M. *et al.* High performance 1.2 Ah Si-alloy/Graphite|LiNi_0.5_Mn_0.3_Co_0.2_O_2_ prototype Li-ion battery. *J. Power Sources* **357**, 188-197 (2017).

19 Son, I. H. *et al.* Silicon carbide-free graphene growth on silicon for lithium-ion battery with high volumetric energy density. *Nat. Commun.* **6,** 7393 (2015).

20 Park, S. H. *et al.* High areal capacity battery electrodes enabled by segregated nanotube networks. *Nat. Energy* **4**, 560-567 (2019).
